# Supplementary material for: Diversity of individual mobility patterns and emergence of aggregated scaling laws
Source: Sci Rep. 2013 Sep 18;3:2678. doi: 10.1038/srep02678 (PMC3776193; doi:10.1038/srep02678)
Supplement: Supplementary Information — Supporting Information [file srep02678-s1.pdf]

Supporting Information
Diversity of Individual Mobility Patterns and Emergence of Aggregated Scaling Laws

Xiao-Yong Yan, Xiao-Pu Han, Bing-Hong Wang, and Tao Zhou

Table S1: Mobility patterns of 230 individuals. In mobility networks, nodes may be too close to be distinguished in geographic view, we therefore draw another version of mobility network in a circular layout by putting the most frequently visited node of a network at the center and ignoring the geolocations.

Individual ID	Displacement distribution	Mobility network (with geolocation)	Mobility network (without geolocation)
ID: 1<br>Status: Housemaker<br>Sex: Female<br>Age: 44	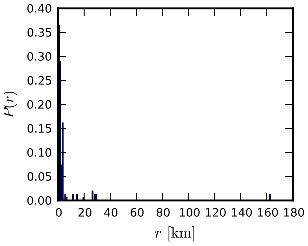	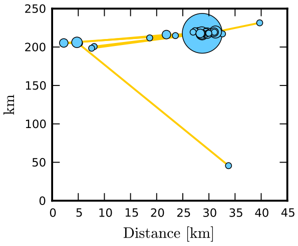	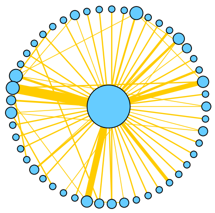
ID: 2<br>Status: Employed<br>Sex: Female<br>Age: 58	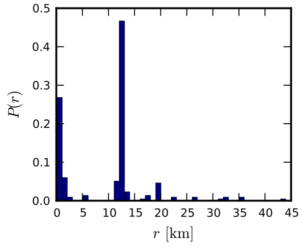	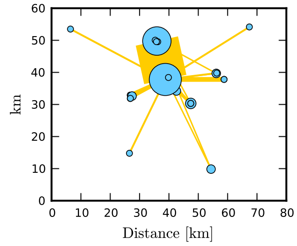	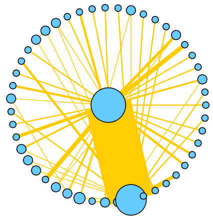
ID: 3<br>Status: Employed<br>Sex: Male<br>Age: 58	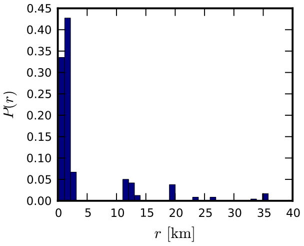	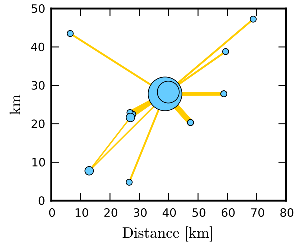	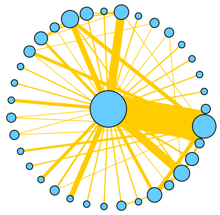
ID: 4<br>Status: Unknow<br>Sex: Female<br>Age: 64	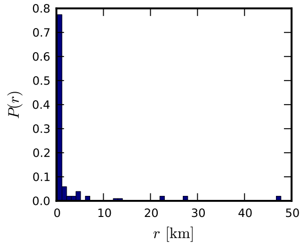	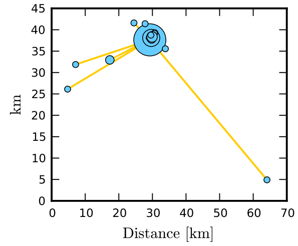	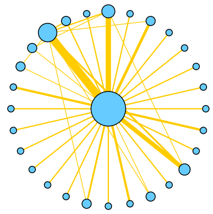

TABLE S1 (continued)

Individual ID	Displacement distribution	Mobility network (with geolocation)	Mobility network (without geolocation)
ID: 5<br>Status: Employed<br>Sex: Male<br>Age: 60	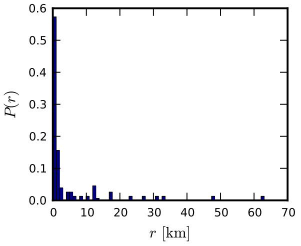	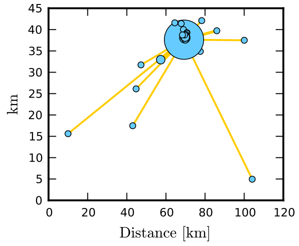	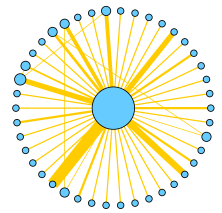
ID: 6<br>Status: Unknow<br>Sex: Male<br>Age: 76	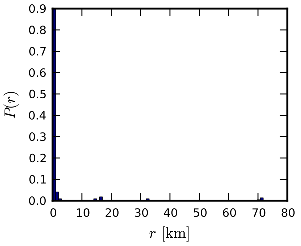	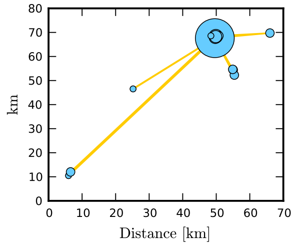	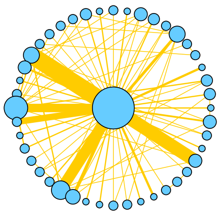
ID: 7<br>Status: Employed<br>Sex: Female<br>Age: 49	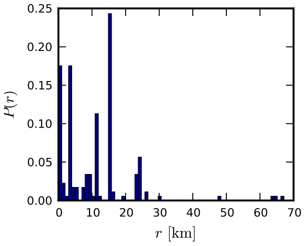	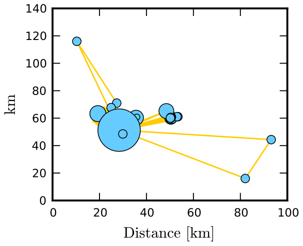	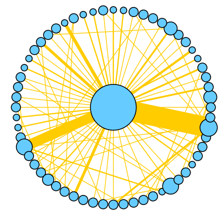
ID: 8<br>Status: Employed<br>Sex: Male<br>Age: 50	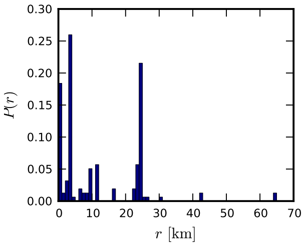	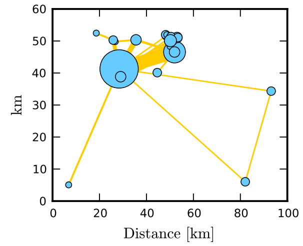	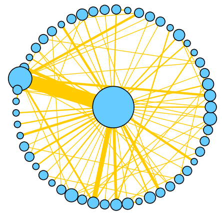
ID: 9<br>Status: Employed<br>Sex: Male<br>Age: 45	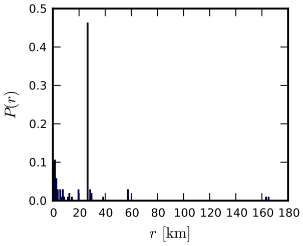	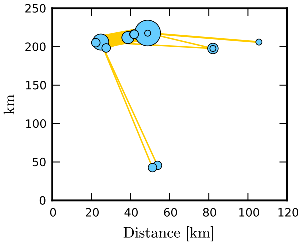	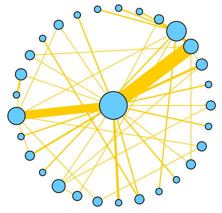
ID: 10<br>Status: Pupil<br>Sex: Female<br>Age: 10	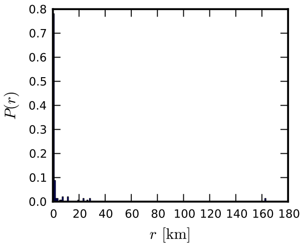	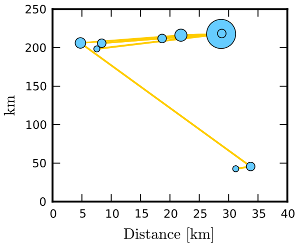	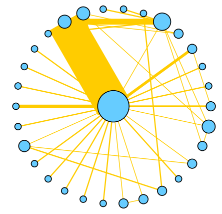

TABLE S1 (continued)

Individual ID	Displacement distribution	Mobility network (with geolocation)	Mobility network (without geolocation)
ID: 11<br>Status: Employed<br>Sex: Male<br>Age: 42	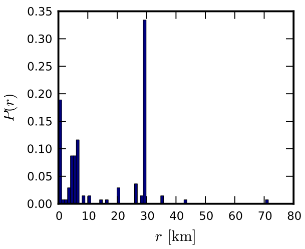	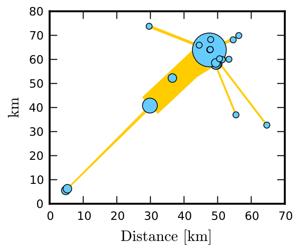	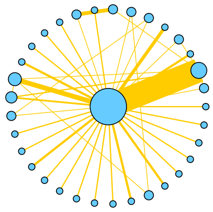
ID: 12<br>Status: Employed<br>Sex: Male<br>Age: 48	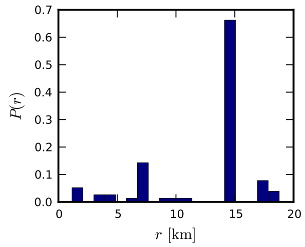	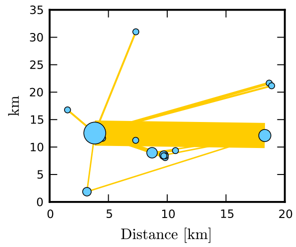	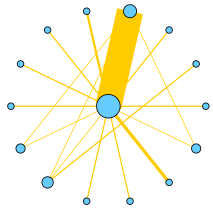
ID: 13<br>Status: Housemaker<br>Sex: Female<br>Age: 42	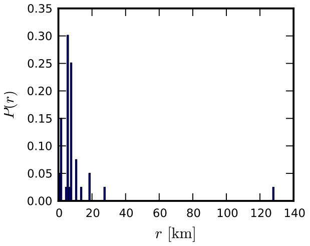	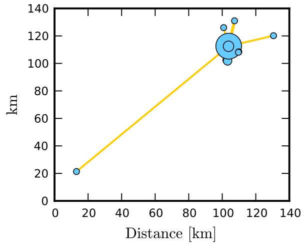	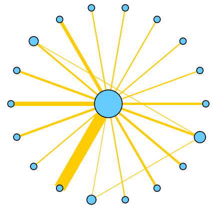
ID: 14<br>Status: Pupil<br>Sex: Male<br>Age: 15	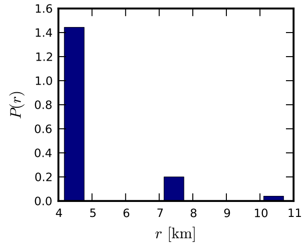	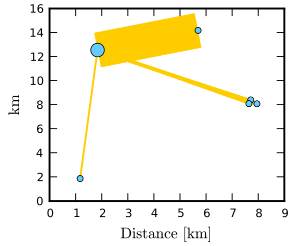	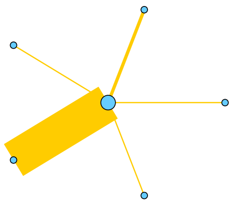
ID: 15<br>Status: Apprentice<br>Sex: Female<br>Age: 16	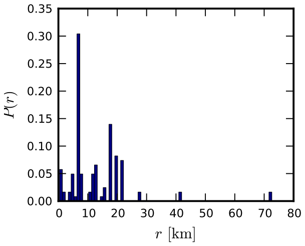	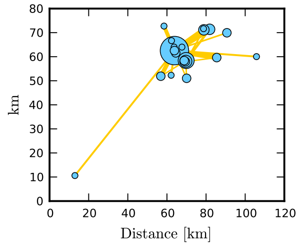	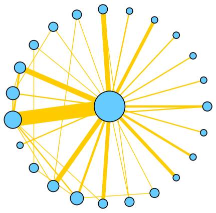
ID: 16<br>Status: Employed<br>Sex: Female<br>Age: 36	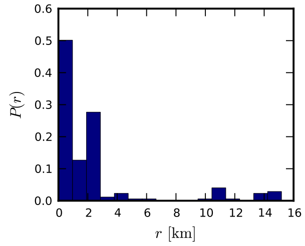	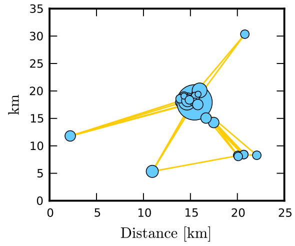	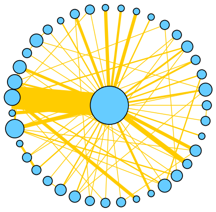

TABLE S1 (continued)

<table>
<tr><th>Individual ID</th><th>Displacement distribution</th><th>Mobility network (with geolocation)</th><th>Mobility network (without geolocation)</th></tr>
<tr><td>ID: 17
Status: Retired
Sex: Female
Age: 67</td><td>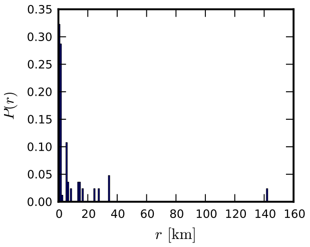
</td><td>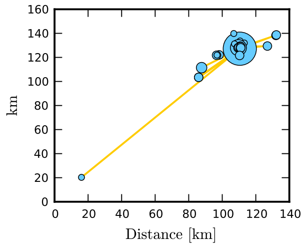
</td><td>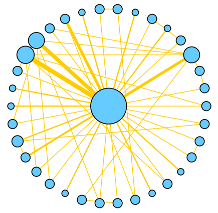</td></tr>
<tr><td>ID: 18
Status: Retired
Sex: Male
Age: 67</td><td>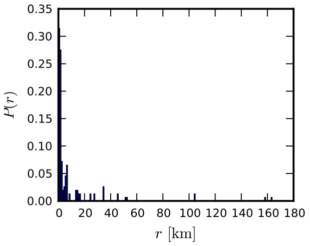
</td><td>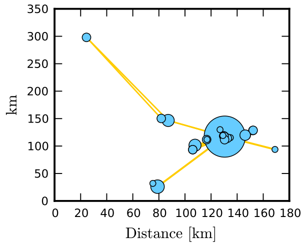
</td><td>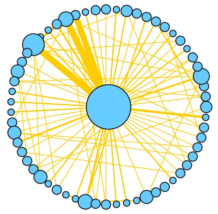</td></tr>
<tr><td>ID: 19
Status: Employed
Sex: Female
Age: 36</td><td>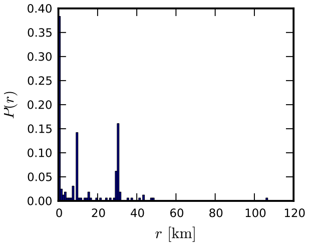
</td><td>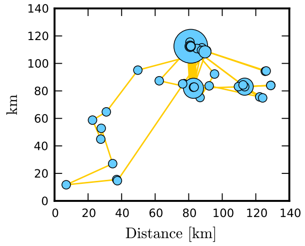
</td><td>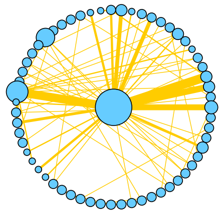</td></tr>
<tr><td>ID: 20
Status: Retired
Sex: Female
Age: 70</td><td>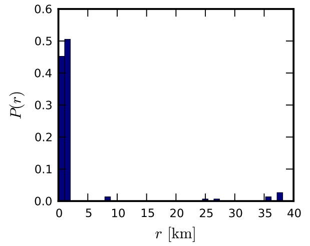
</td><td>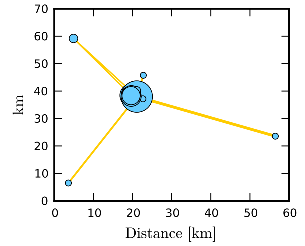
</td><td>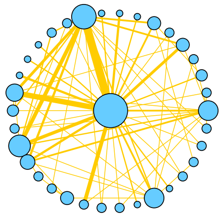</td></tr>
<tr><td>ID: 21
Status: Apprentice
Sex: Female
Age: 17</td><td>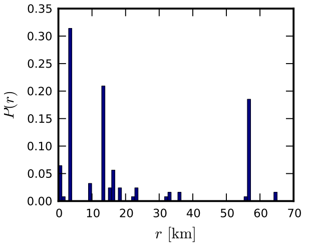
</td><td>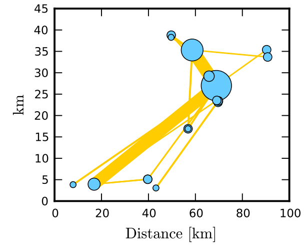
</td><td>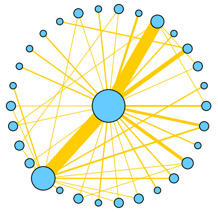</td></tr>
<tr><td>ID: 22
Status: Unknow
Sex: Female
Age: 43</td><td>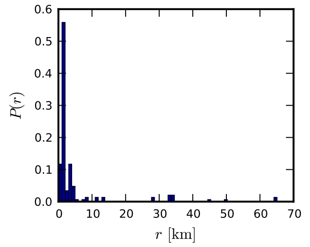
</td><td>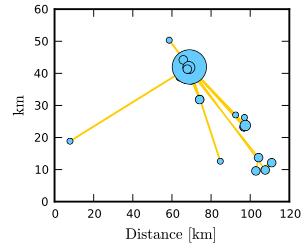
</td><td>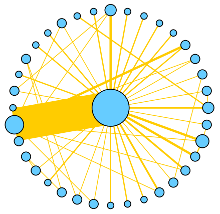</td></tr>
</table>

TABLE S1 (continued)

Individual ID	Displacement distribution	Mobility network (with geolocation)	Mobility network (without geolocation)
ID: 23<br>Status: Employed<br>Sex: Male<br>Age: 31	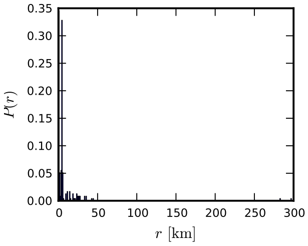	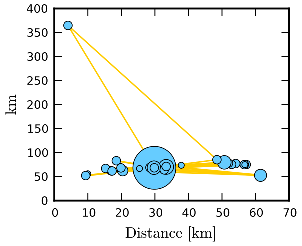	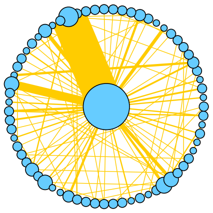
ID: 24<br>Status: Student<br>Sex: Female<br>Age: 30	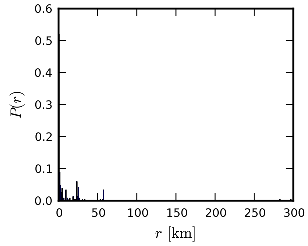	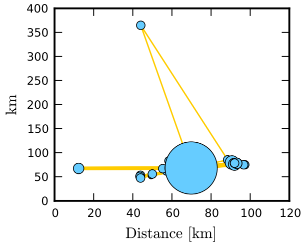	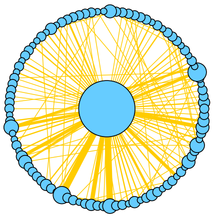
ID: 25<br>Status: Employed<br>Sex: Male<br>Age: 46	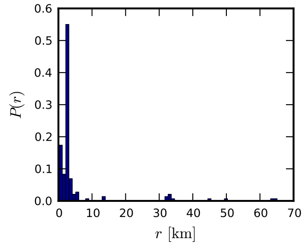	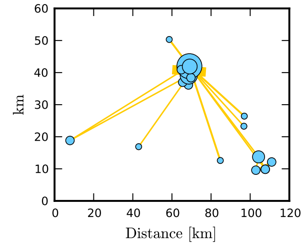	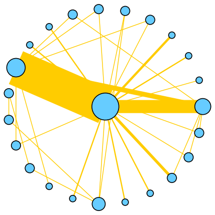
ID: 26<br>Status: Retired<br>Sex: Male<br>Age: 68	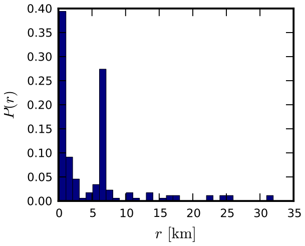	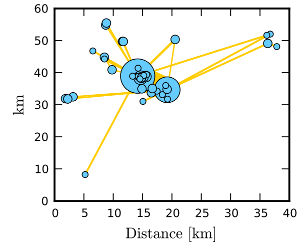	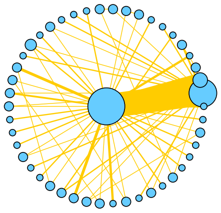
ID: 27<br>Status: Employed<br>Sex: Male<br>Age: 51	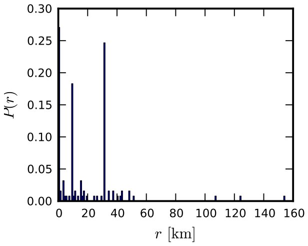	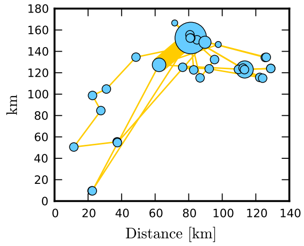	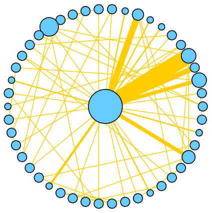
ID: 28<br>Status: Employed<br>Sex: Male<br>Age: 32	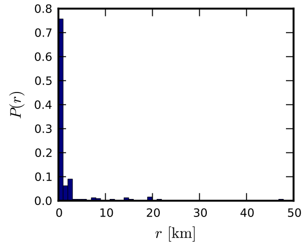	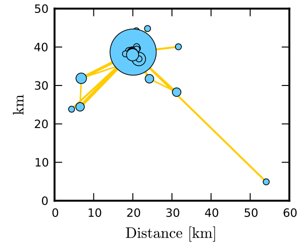	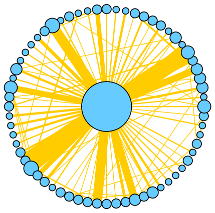

TABLE S1 (continued)

Individual ID	Displacement distribution	Mobility network (with geolocation)	Mobility network (without geolocation)
ID: 29<br>Status: Retired<br>Sex: Male<br>Age: 76	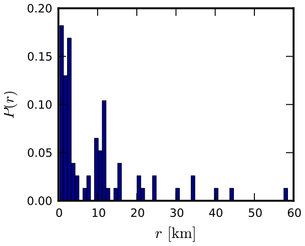	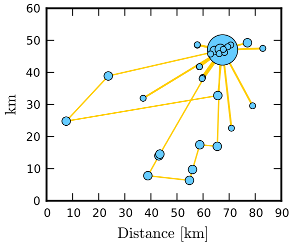	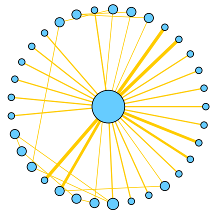
ID: 30<br>Status: Employed<br>Sex: Male<br>Age: 53	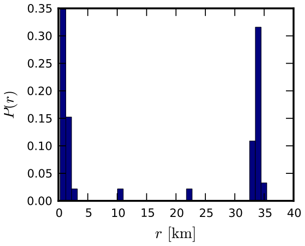	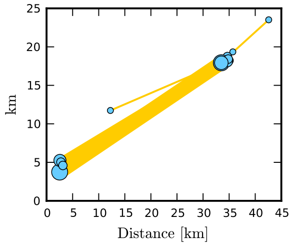	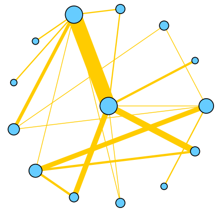
ID: 31<br>Status: Retired<br>Sex: Female<br>Age: 70	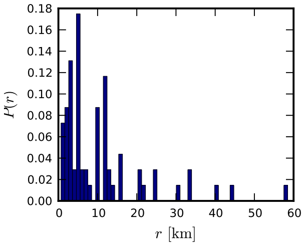	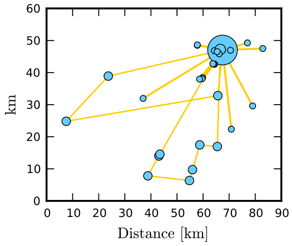	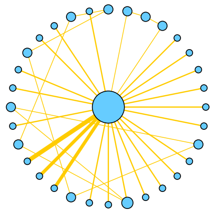
ID: 32<br>Status: Employed<br>Sex: Female<br>Age: 52	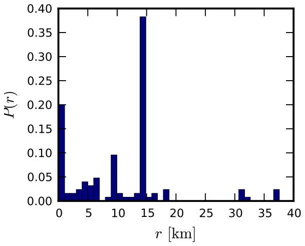	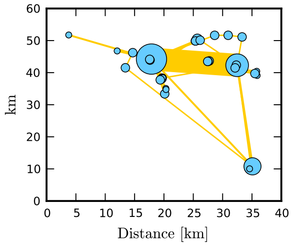	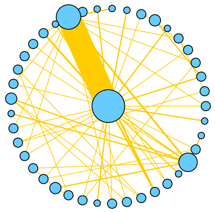
ID: 33<br>Status: Retired<br>Sex: Male<br>Age: 60	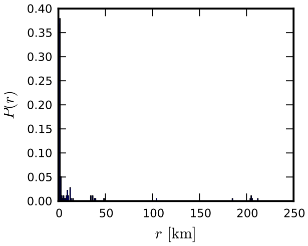	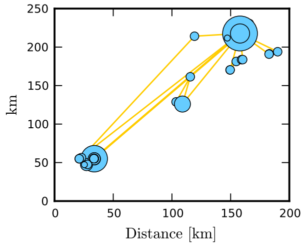	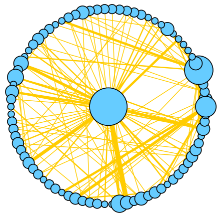
ID: 34<br>Status: Retired<br>Sex: Female<br>Age: 57	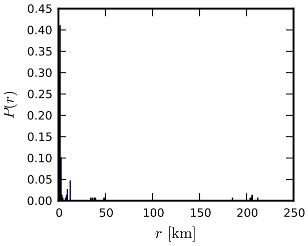	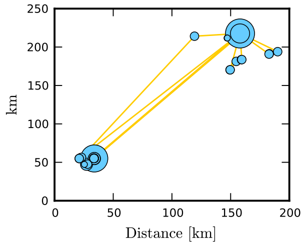	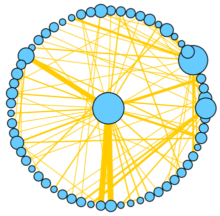

TABLE S1 (continued)

Individual ID	Displacement distribution	Mobility network (with geolocation)	Mobility network (without geolocation)
ID: 35<br>Status: Employed<br>Sex: Female<br>Age: 39	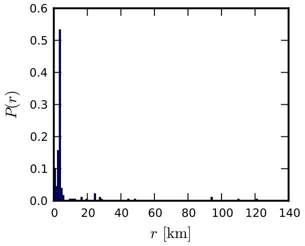	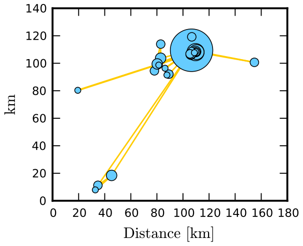	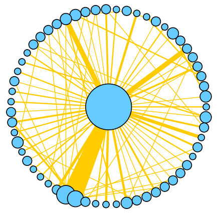
ID: 36<br>Status: Employed<br>Sex: Male<br>Age: 47	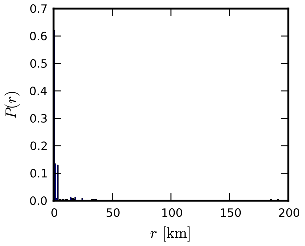	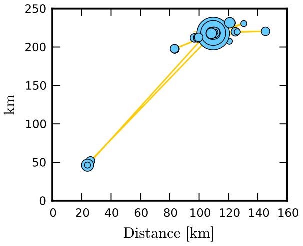	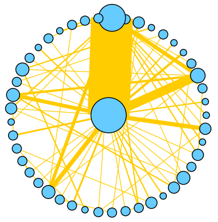
ID: 37<br>Status: Employed<br>Sex: Female<br>Age: 31	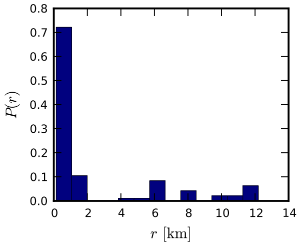	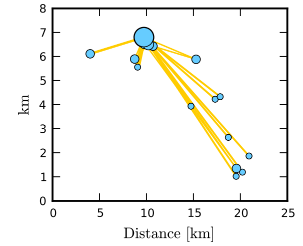	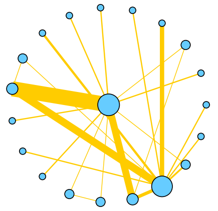
ID: 38<br>Status: Employed<br>Sex: Male<br>Age: 32	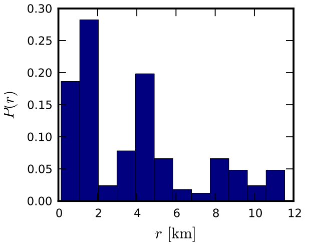	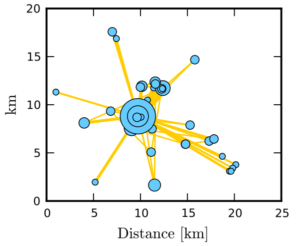	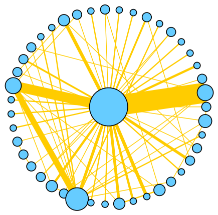
ID: 39<br>Status: Employed<br>Sex: Male<br>Age: 56	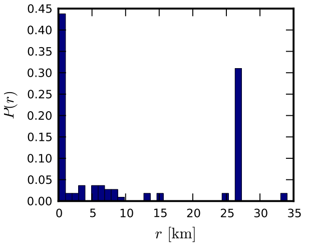	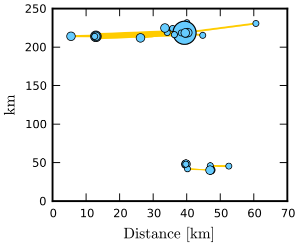	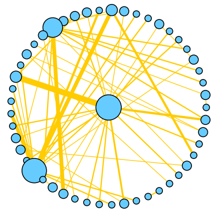
ID: 40<br>Status: Student<br>Sex: Female<br>Age: 20	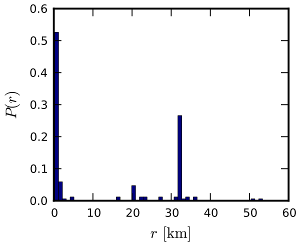	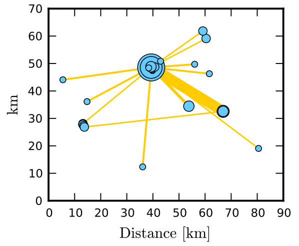	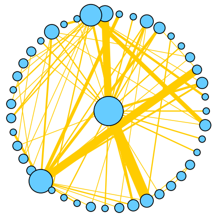

TABLE S1 (continued)

Individual ID	Displacement distribution	Mobility network (with geolocation)	Mobility network (without geolocation)
ID: 41<br>Status: Employed<br>Sex: Female<br>Age: 23	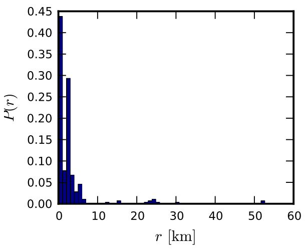	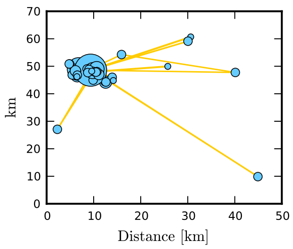	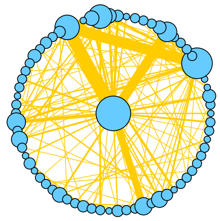
ID: 42<br>Status: Unknow<br>Sex: Male<br>Age: 37	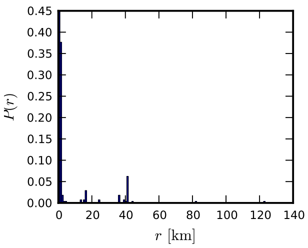	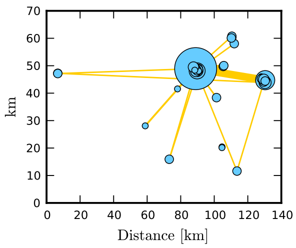	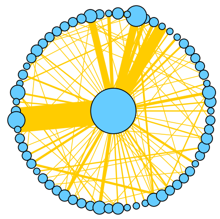
ID: 43<br>Status: Unknow<br>Sex: Female<br>Age: 35	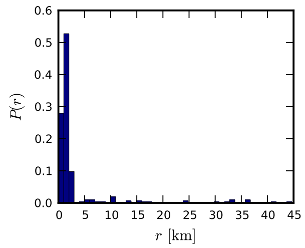	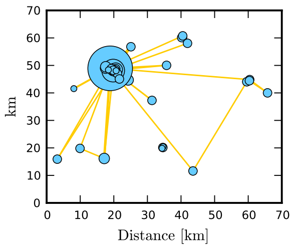	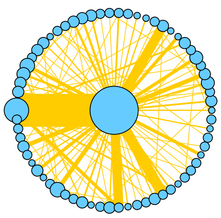
ID: 44<br>Status: Unknow<br>Sex: Female<br>Age: 62	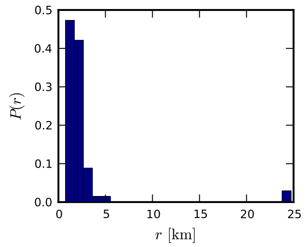	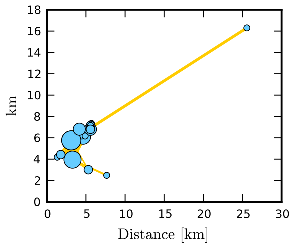	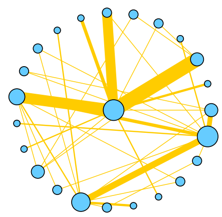
ID: 45<br>Status: Student<br>Sex: Male<br>Age: 23	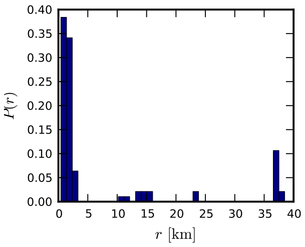	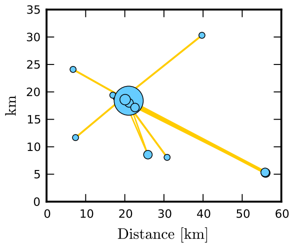	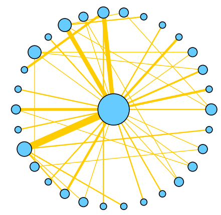
ID: 46<br>Status: Employed<br>Sex: Male<br>Age: 61	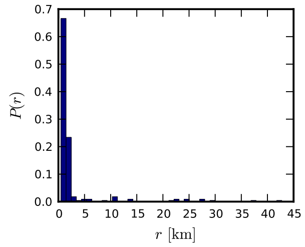	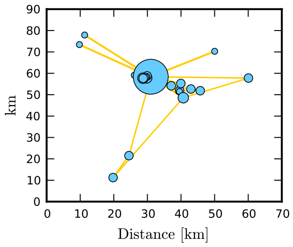	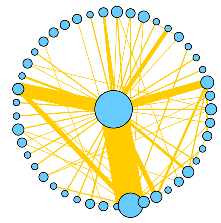

TABLE S1 (continued)

Individual ID	Displacement distribution	Mobility network (with geolocation)	Mobility network (without geolocation)
ID: 47<br>Status: Pupil<br>Sex: Female<br>Age: 10	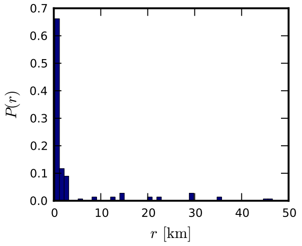	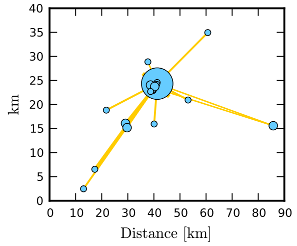	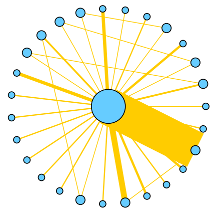
ID: 48<br>Status: Employed<br>Sex: Male<br>Age: 32	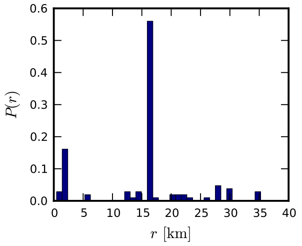	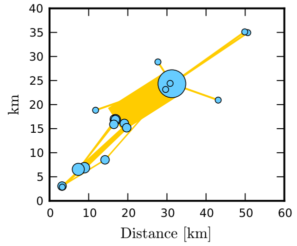	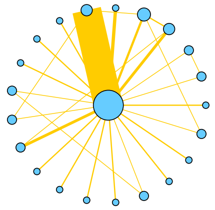
ID: 49<br>Status: Housemaker<br>Sex: Female<br>Age: 37	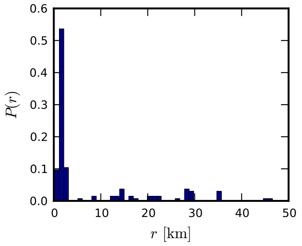	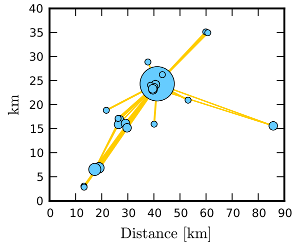	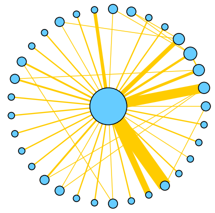
ID: 50<br>Status: Employed<br>Sex: Male<br>Age: 60	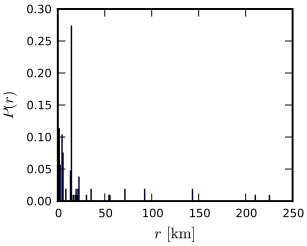	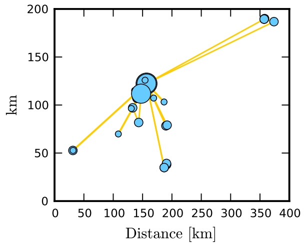	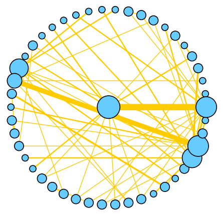
ID: 51<br>Status: Employed<br>Sex: Female<br>Age: 53	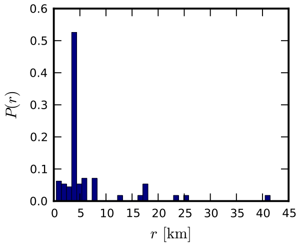	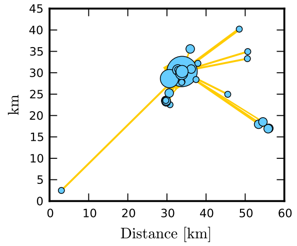	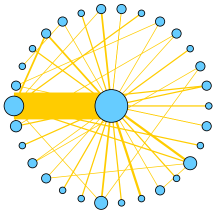
ID: 52<br>Status: Retired<br>Sex: Male<br>Age: 72	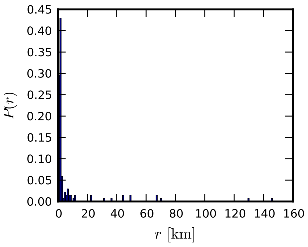	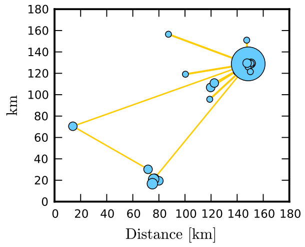	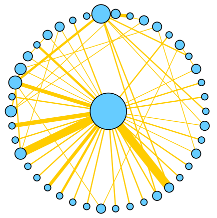

TABLE S1 (continued)

Individual ID	Displacement distribution	Mobility network (with geolocation)	Mobility network (without geolocation)
ID: 53<br>Status: Retired<br>Sex: Female<br>Age: 70	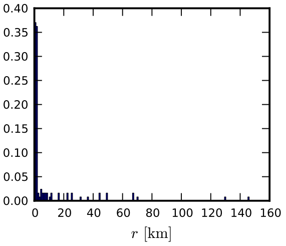	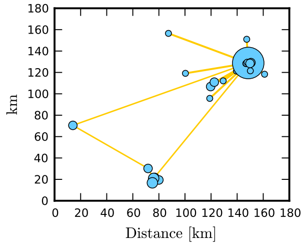	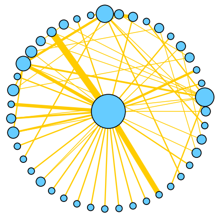
ID: 54<br>Status: Employed<br>Sex: Male<br>Age: 62	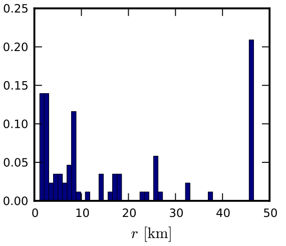	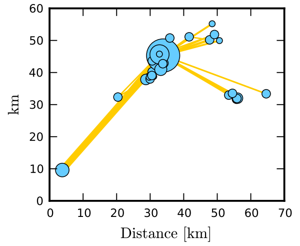	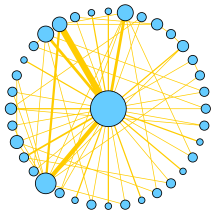
ID: 55<br>Status: Employed<br>Sex: Male<br>Age: 53	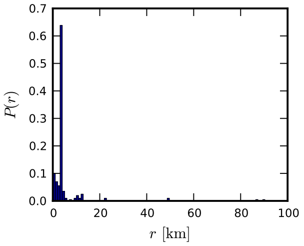	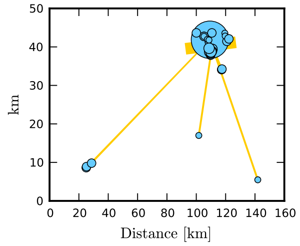	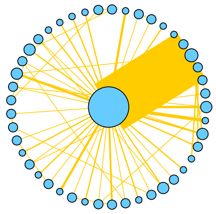
ID: 56<br>Status: Retired<br>Sex: Female<br>Age: 64	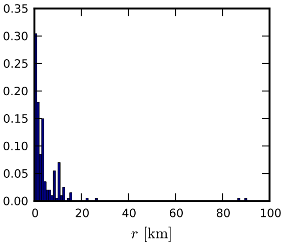	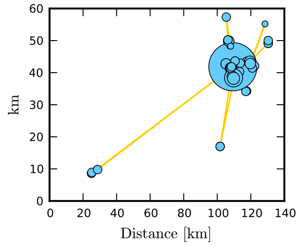	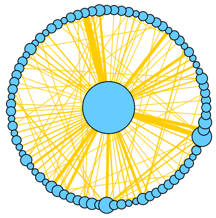
ID: 57<br>Status: Pupil<br>Sex: Male<br>Age: 12	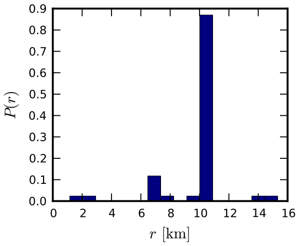	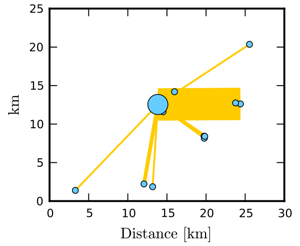	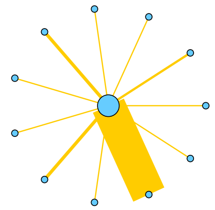
ID: 58<br>Status: Housemaker<br>Sex: Female<br>Age: 69	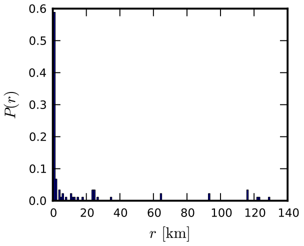	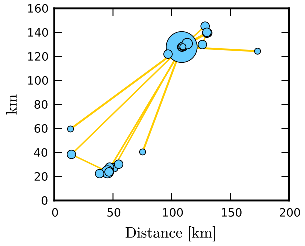	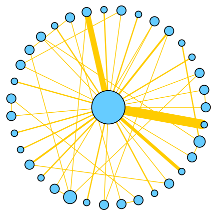

TABLE S1 (continued)

<table>
<tr><th>Individual ID</th><th>Displacement distribution</th><th>Mobility network (with geolocation)</th><th>Mobility network (without geolocation)</th></tr>
<tr><td>ID: 59
Status: Employed
Sex: Male
Age: 57</td><td>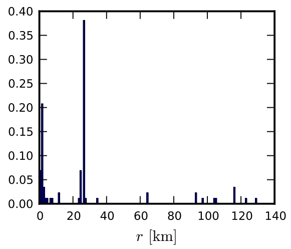</td><td>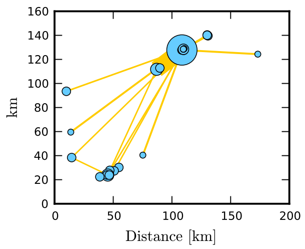</td><td>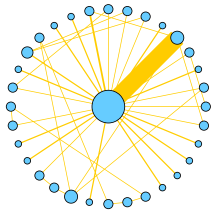</td></tr>
<tr><td>ID: 60
Status: Employed
Sex: Male
Age: 56</td><td>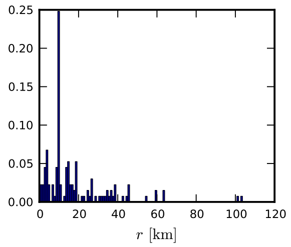</td><td>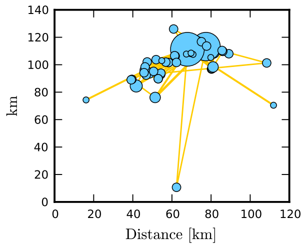</td><td>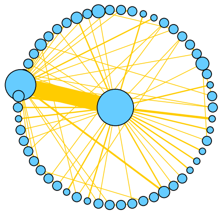</td></tr>
<tr><td>ID: 61
Status: Housemaker
Sex: Female
Age: 54</td><td>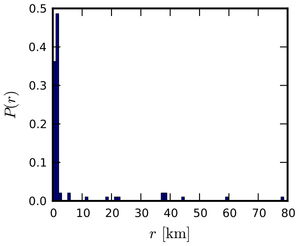</td><td>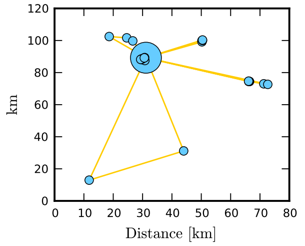</td><td>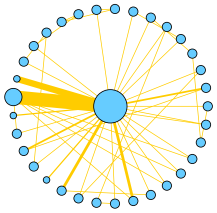</td></tr>
<tr><td>ID: 62
Status: Employed
Sex: Male
Age: 49</td><td>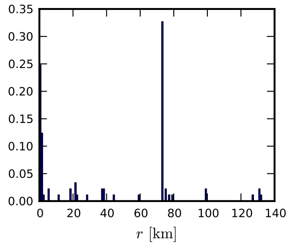</td><td>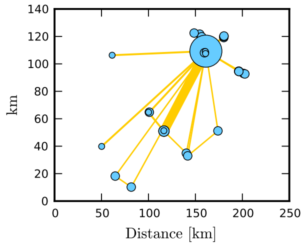</td><td>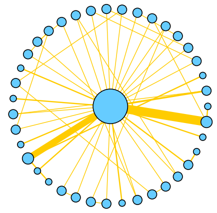</td></tr>
<tr><td>ID: 63
Status: Pupil
Sex: Male
Age: 15</td><td>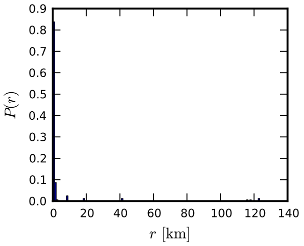</td><td>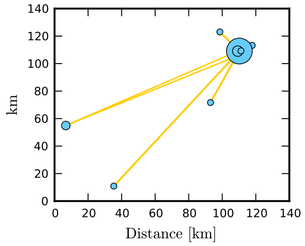</td><td>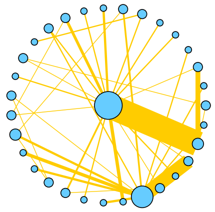</td></tr>
<tr><td>ID: 64
Status: Unknow
Sex: Male
Age: 47</td><td>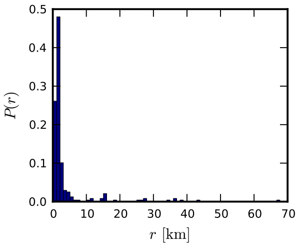</td><td>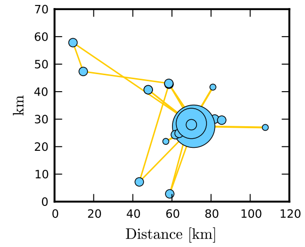</td><td>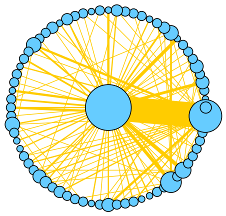</td></tr>
</table>

TABLE S1 (continued)

Individual ID	Displacement distribution	Mobility network (with geolocation)	Mobility network (without geolocation)
ID: 65<br>Status: Pupil<br>Sex: Male<br>Age: 12	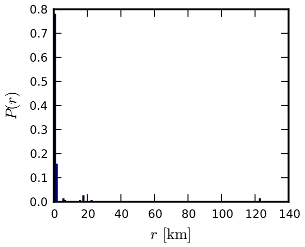	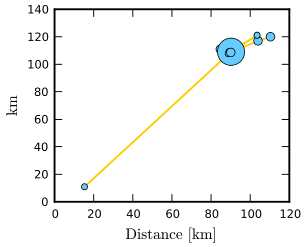	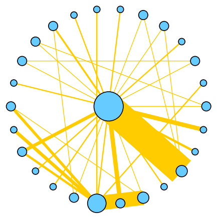
ID: 66<br>Status: Pupil<br>Sex: Female<br>Age: 13	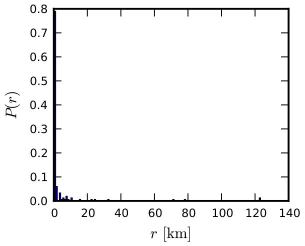	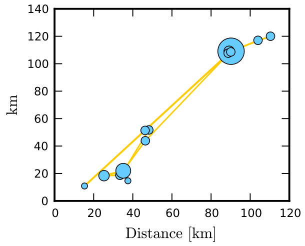	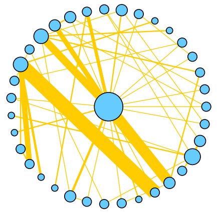
ID: 67<br>Status: Apprentice<br>Sex: Female<br>Age: 16	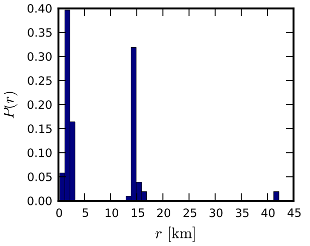	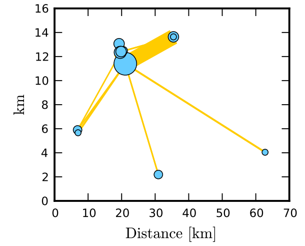	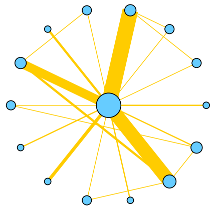
ID: 68<br>Status: Retired<br>Sex: Male<br>Age: 66	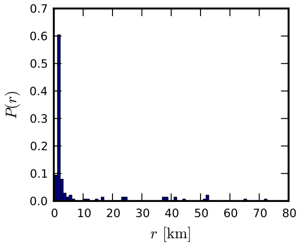	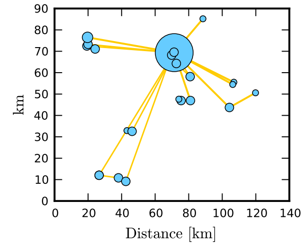	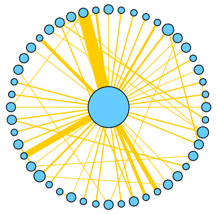
ID: 69<br>Status: Housemaker<br>Sex: Female<br>Age: 57	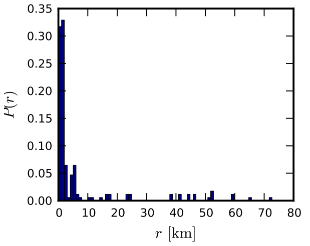	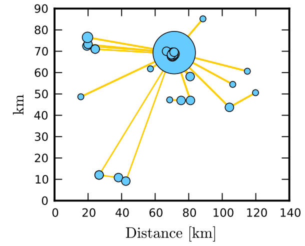	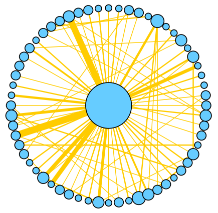
ID: 70<br>Status: Unknow<br>Sex: Male<br>Age: 33	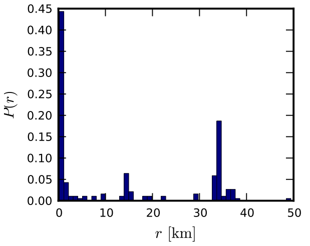	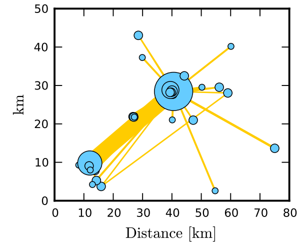	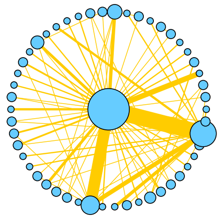

TABLE S1 (continued)

Individual ID	Displacement distribution	Mobility network (with geolocation)	Mobility network (without geolocation)
ID: 71<br>Status: Unknow<br>Sex: Female<br>Age: 28	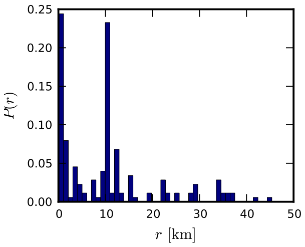	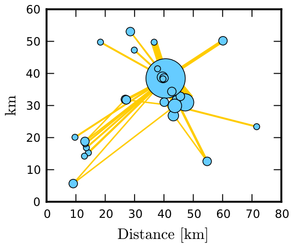	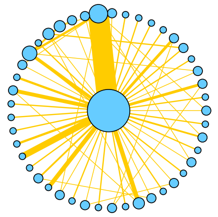
ID: 72<br>Status: Retired<br>Sex: Male<br>Age: 68	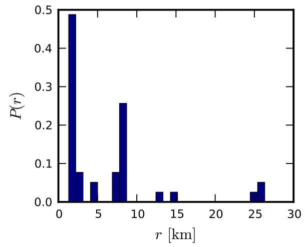	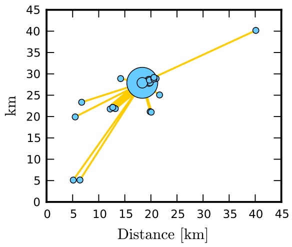	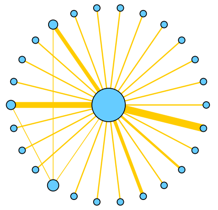
ID: 73<br>Status: Retired<br>Sex: Female<br>Age: 68	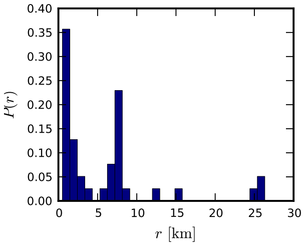	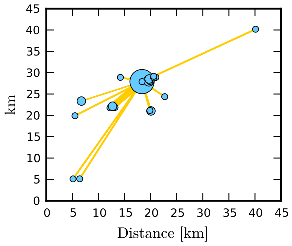	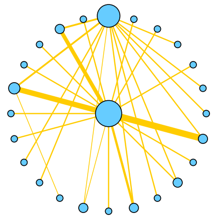
ID: 74<br>Status: Employed<br>Sex: Male<br>Age: 46	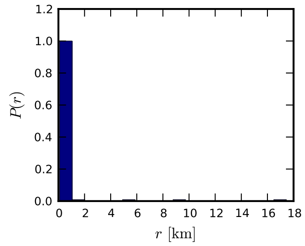	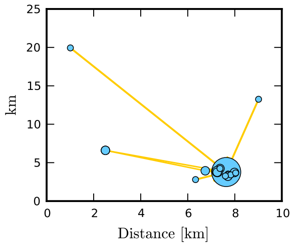	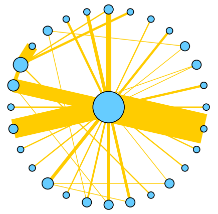
ID: 75<br>Status: Employed<br>Sex: Female<br>Age: 55	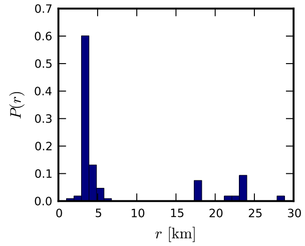	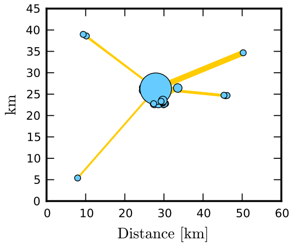	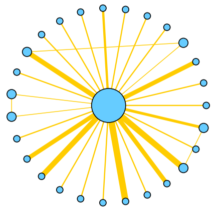
ID: 76<br>Status: Housemaker<br>Sex: Female<br>Age: 46	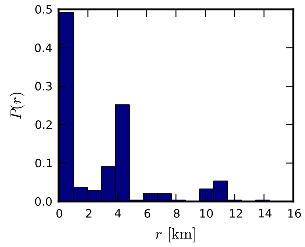	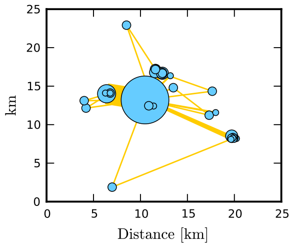	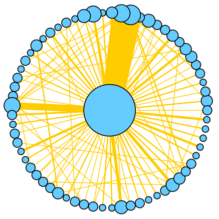

TABLE S1 (continued)

Individual ID	Displacement distribution	Mobility network (with geolocation)	Mobility network (without geolocation)
ID: 77<br>Status: Employed<br>Sex: Male<br>Age: 48	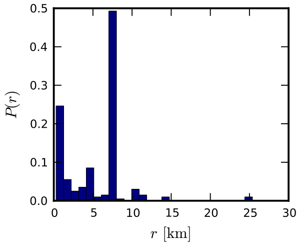	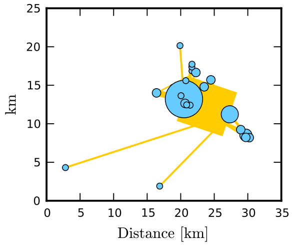	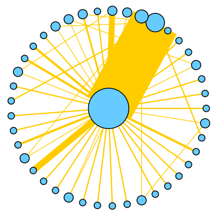
ID: 78<br>Status: Pupil<br>Sex: Female<br>Age: 10	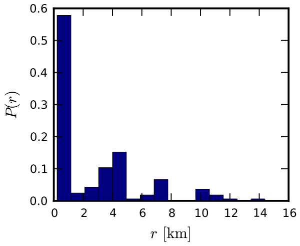	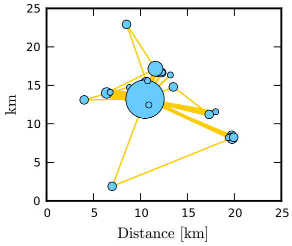	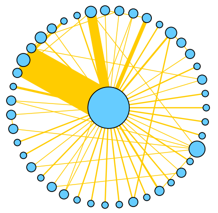
ID: 79<br>Status: Pupil<br>Sex: Female<br>Age: 15	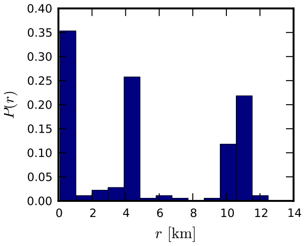	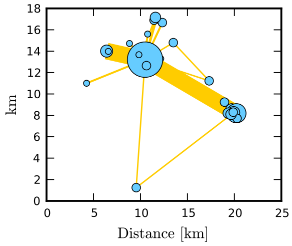	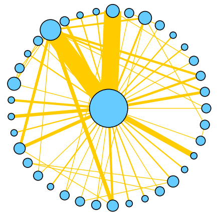
ID: 80<br>Status: Unemployed<br>Sex: Female<br>Age: 51	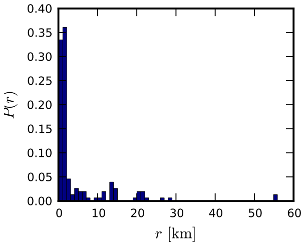	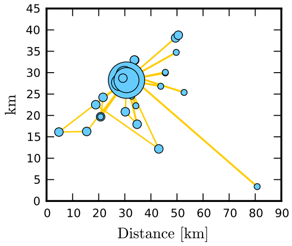	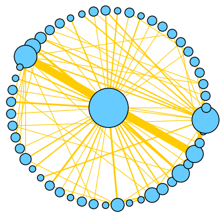
ID: 81<br>Status: Pupil<br>Sex: Male<br>Age: 16	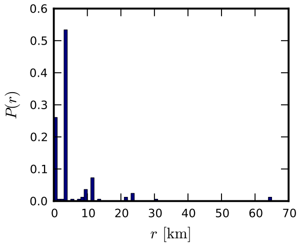	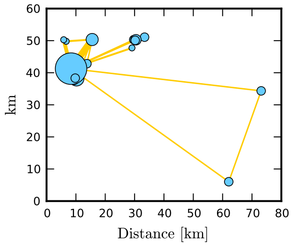	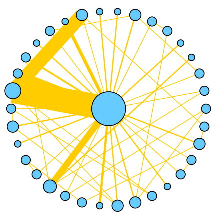
ID: 82<br>Status: Pupil<br>Sex: Male<br>Age: 13	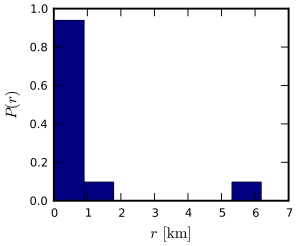	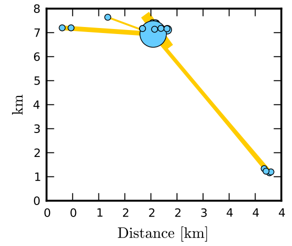	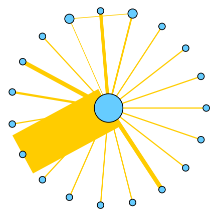

TABLE S1 (continued)

Individual ID	Displacement distribution	Mobility network (with geolocation)	Mobility network (without geolocation)
ID: 83<br>Status: Pupil<br>Sex: Male<br>Age: 14	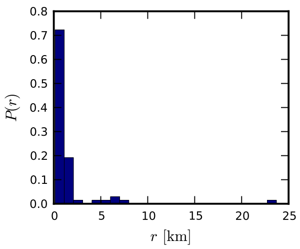	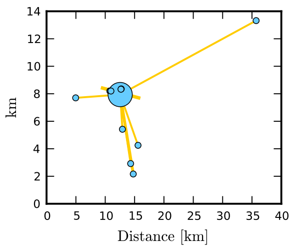	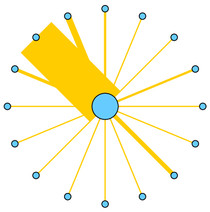
ID: 84<br>Status: Pupil<br>Sex: Male<br>Age: 14	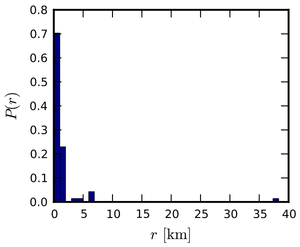	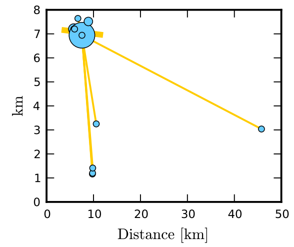	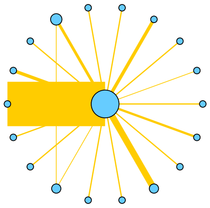
ID: 85<br>Status: Pupil<br>Sex: Female<br>Age: 10	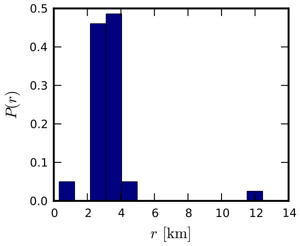	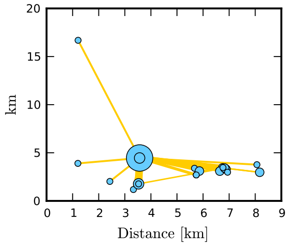	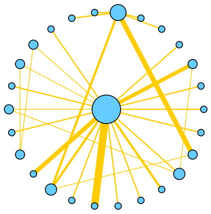
ID: 86<br>Status: Pupil<br>Sex: Female<br>Age: 12	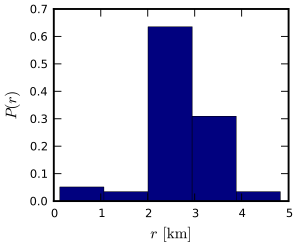	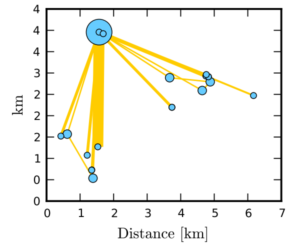	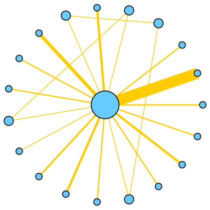
ID: 87<br>Status: Pupil<br>Sex: Female<br>Age: 14	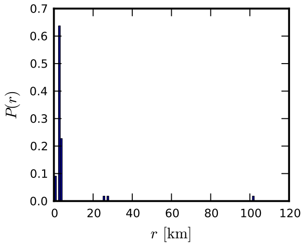	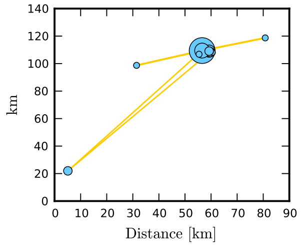	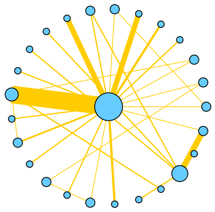
ID: 88<br>Status: Employed<br>Sex: Male<br>Age: 41	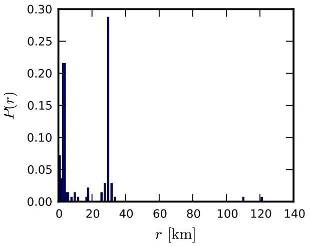	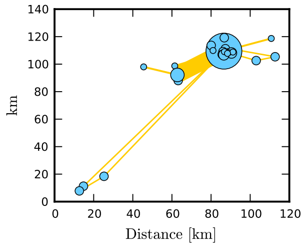	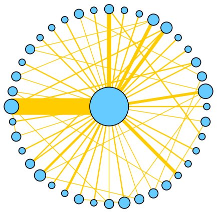

TABLE S1 (continued)

Individual ID	Displacement distribution	Mobility network (with geolocation)	Mobility network (without geolocation)
ID: 89<br>Status: Employed<br>Sex: Male<br>Age: 41	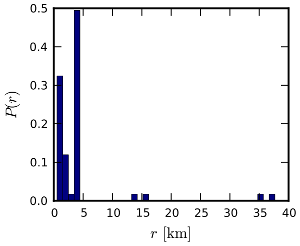	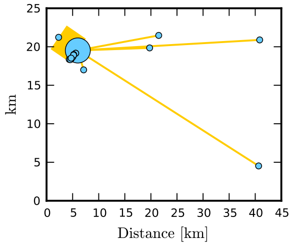	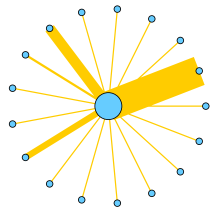
ID: 90<br>Status: Apprentice<br>Sex: Male<br>Age: 18	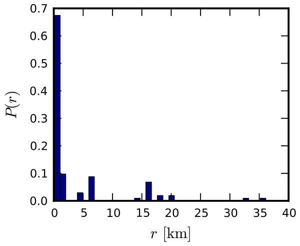	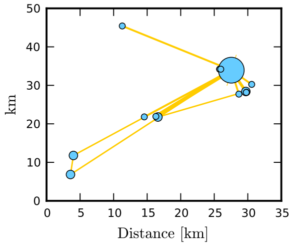	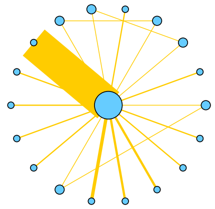
ID: 91<br>Status: Employed<br>Sex: Male<br>Age: 58	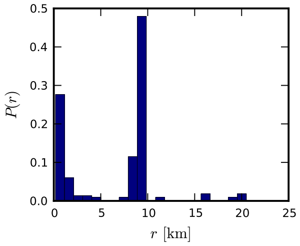	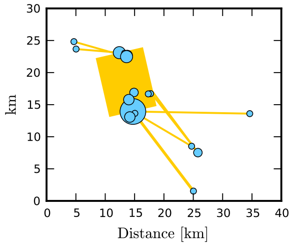	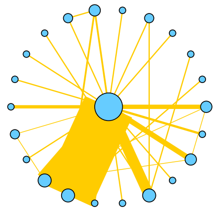
ID: 92<br>Status: Housemaker<br>Sex: Female<br>Age: 57	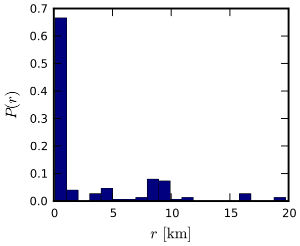	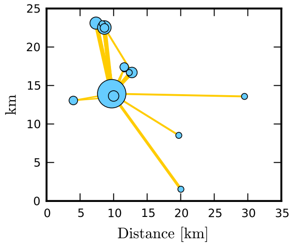	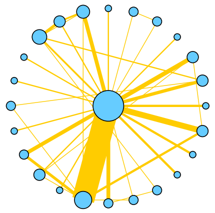
ID: 93<br>Status: Unknow<br>Sex: Female<br>Age: 55	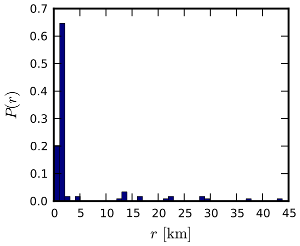	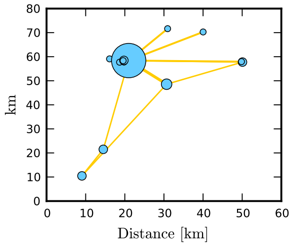	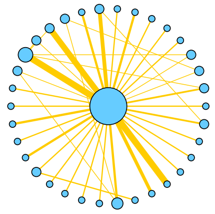
ID: 94<br>Status: Employed<br>Sex: Female<br>Age: 55	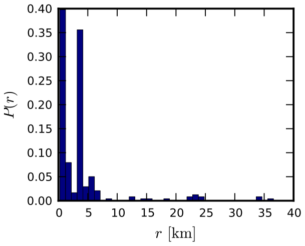	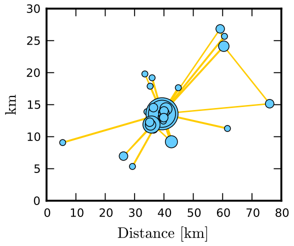	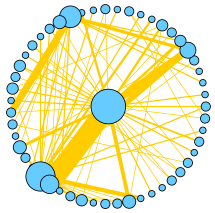

TABLE S1 (continued)

Individual ID	Displacement distribution	Mobility network (with geolocation)	Mobility network (without geolocation)
ID: 95<br>Status: Pupil<br>Sex: Female<br>Age: 17	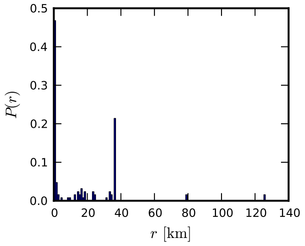	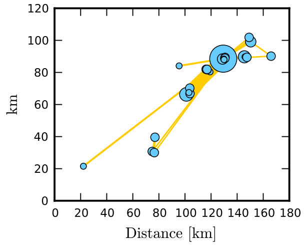	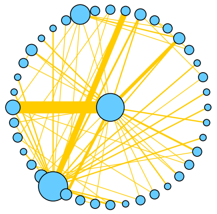
ID: 96<br>Status: Employed<br>Sex: Female<br>Age: 51	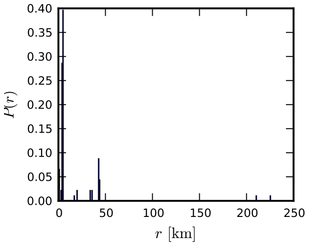	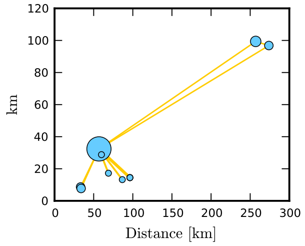	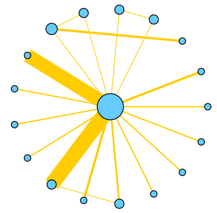
ID: 97<br>Status: Pupil<br>Sex: Male<br>Age: 15	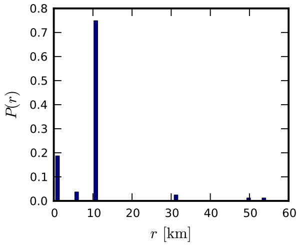	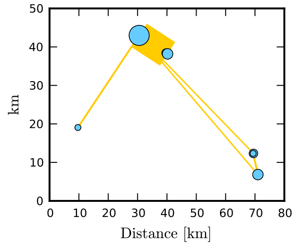	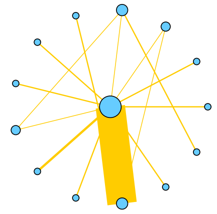
ID: 98<br>Status: Employed<br>Sex: Male<br>Age: 47	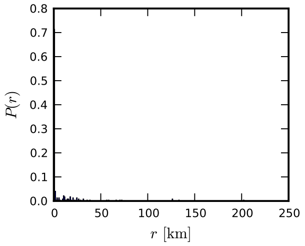	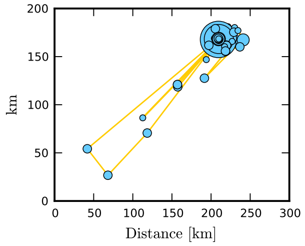	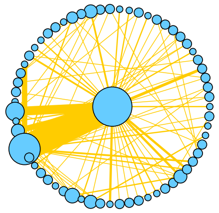
ID: 99<br>Status: Housemaker<br>Sex: Female<br>Age: 40	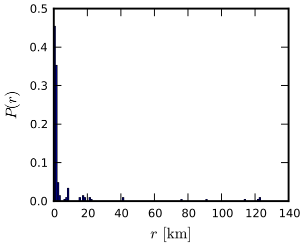	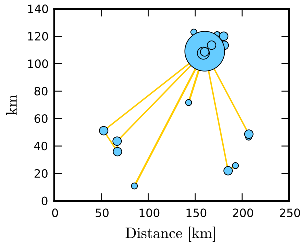	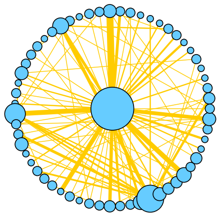
ID: 100<br>Status: Employed<br>Sex: Male<br>Age: 40	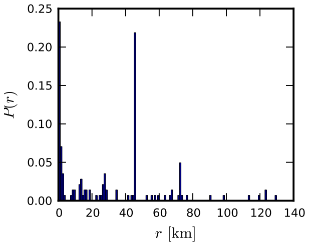	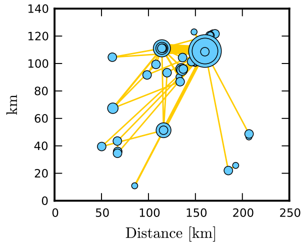	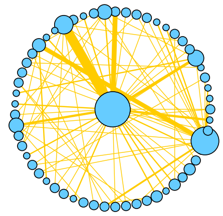

TABLE S1 (continued)

Individual ID	Displacement distribution	Mobility network (with geolocation)	Mobility network (without geolocation)
ID: 101<br>Status: Employed<br>Sex: Male<br>Age: 22	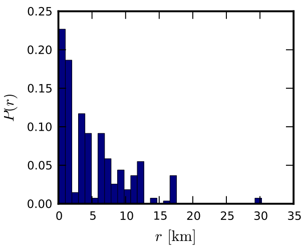	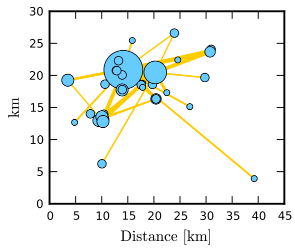	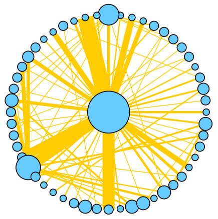
ID: 102<br>Status: Pupil<br>Sex: Female<br>Age: 12	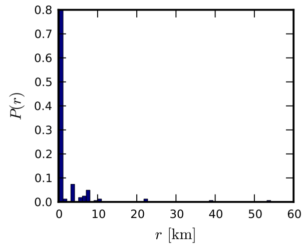	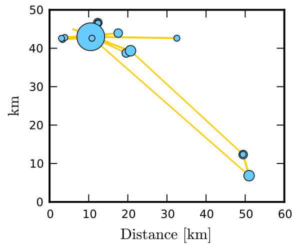	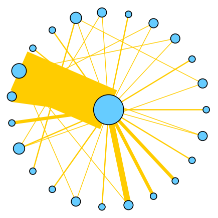
ID: 103<br>Status: Housemaker<br>Sex: Female<br>Age: 45	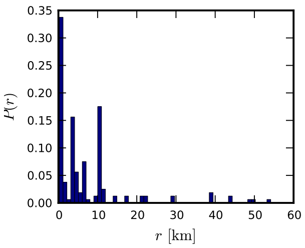	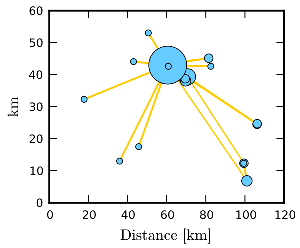	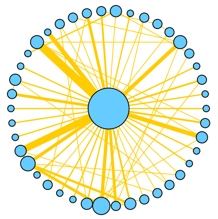
ID: 104<br>Status: Employed<br>Sex: Male<br>Age: 46	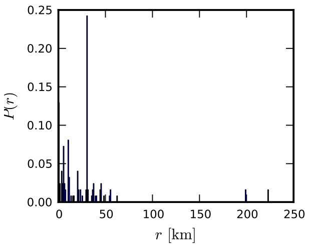	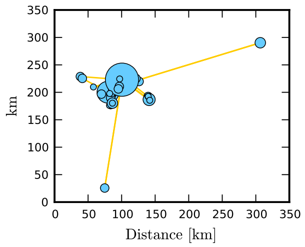	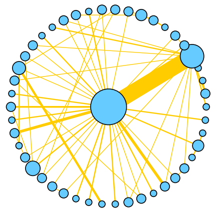
ID: 105<br>Status: Pupil<br>Sex: Male<br>Age: 13	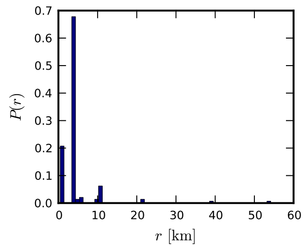	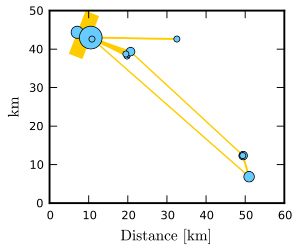	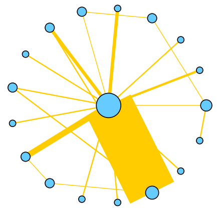
ID: 106<br>Status: Employed<br>Sex: Male<br>Age: 51	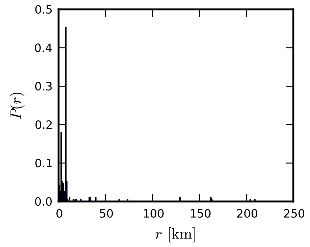	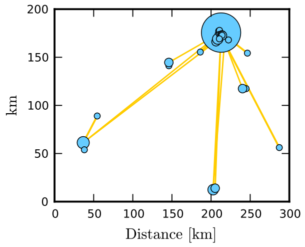	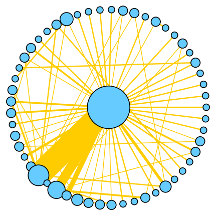

TABLE S1 (continued)

Individual ID	Displacement distribution	Mobility network (with geolocation)	Mobility network (without geolocation)
ID: 107<br>Status: Employed<br>Sex: Female<br>Age: 49	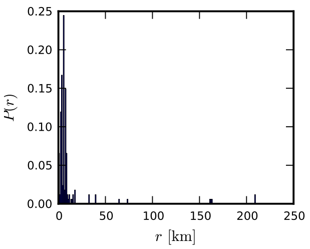	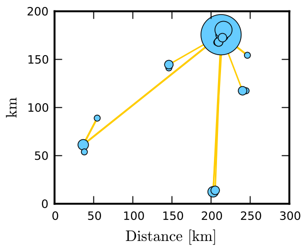	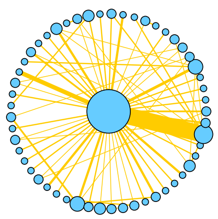
ID: 108<br>Status: Employed<br>Sex: Male<br>Age: 59	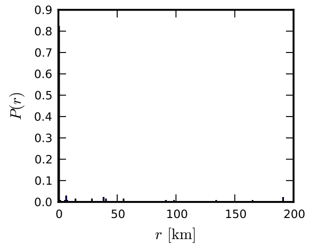	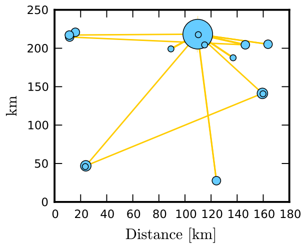	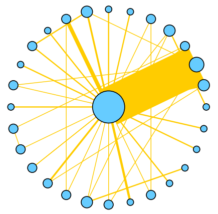
ID: 109<br>Status: Employed<br>Sex: Male<br>Age: 60	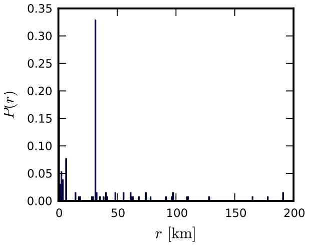	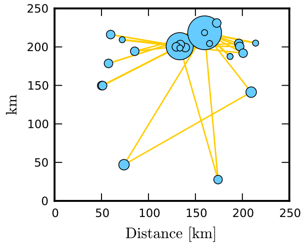	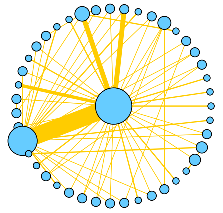
ID: 110<br>Status: Pupil<br>Sex: Female<br>Age: 15	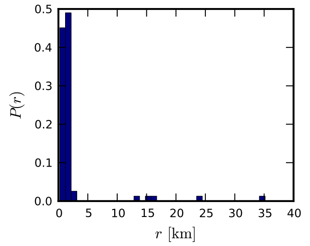	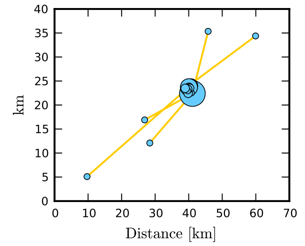	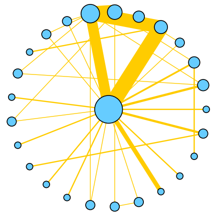
ID: 111<br>Status: Employed<br>Sex: Female<br>Age: 28	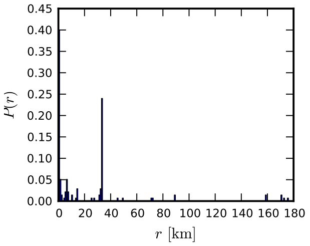	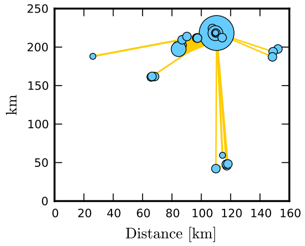	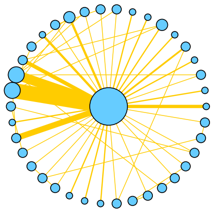
ID: 112<br>Status: Employed<br>Sex: Male<br>Age: 33	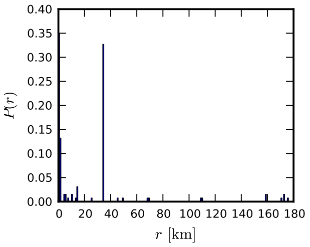	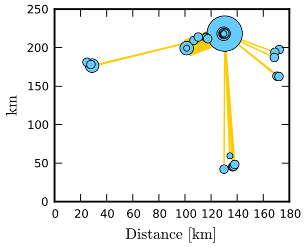	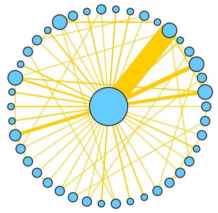

TABLE S1 (continued)

Individual ID	Displacement distribution	Mobility network (with geolocation)	Mobility network (without geolocation)
ID: 113<br>Status: Employed<br>Sex: Male<br>Age: 45	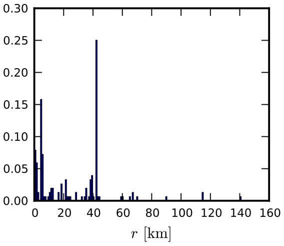	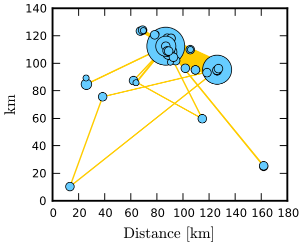	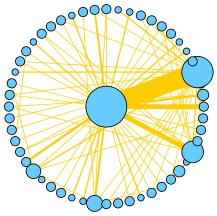
ID: 114<br>Status: Employed<br>Sex: Male<br>Age: 42	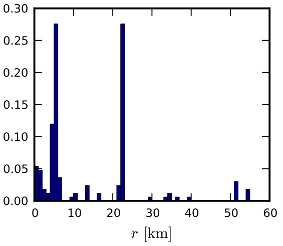	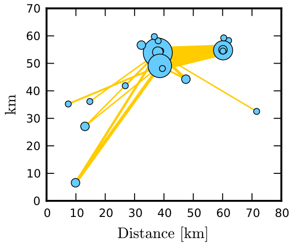	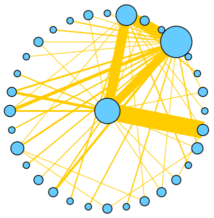
ID: 115<br>Status: Employed<br>Sex: Male<br>Age: 43	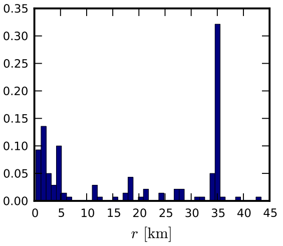	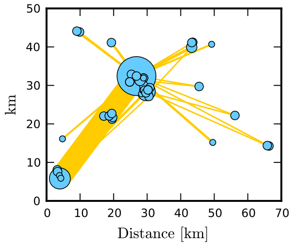	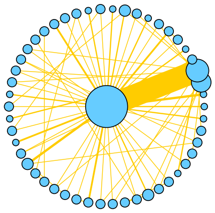
ID: 116<br>Status: Employed<br>Sex: Female<br>Age: 45	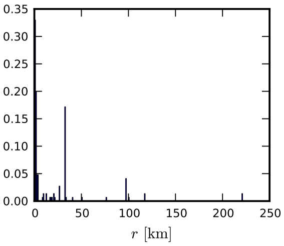	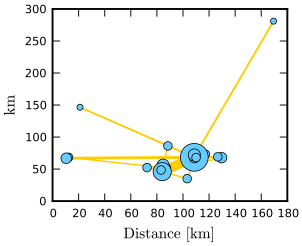	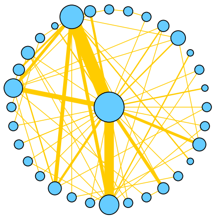
ID: 117<br>Status: Unknow<br>Sex: Female<br>Age: 69	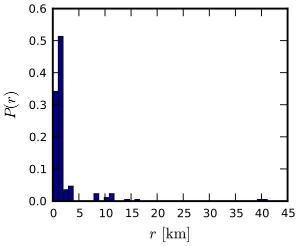	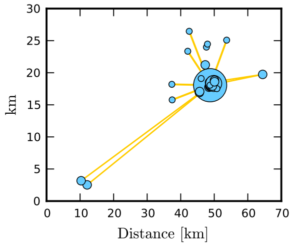	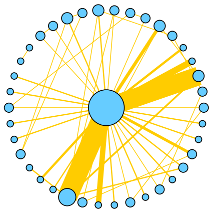
ID: 118<br>Status: Unknow<br>Sex: Male<br>Age: 73	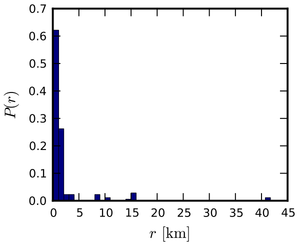	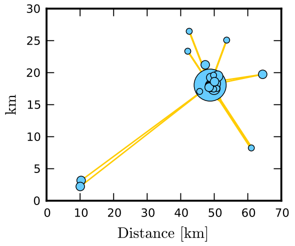	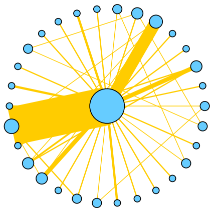

TABLE S1 (continued)

Individual ID	Displacement distribution	Mobility network (with geolocation)	Mobility network (without geolocation)
ID: 119<br>Status: Employed<br>Sex: Female<br>Age: 40	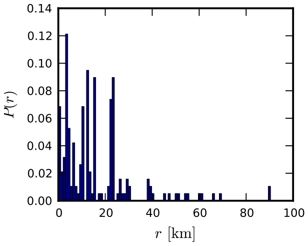	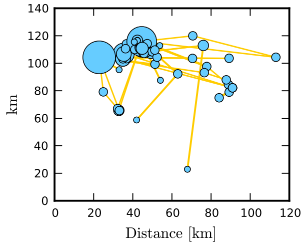	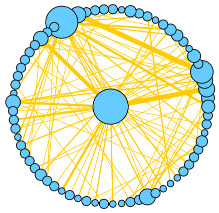
ID: 120<br>Status: Employed<br>Sex: Male<br>Age: 55	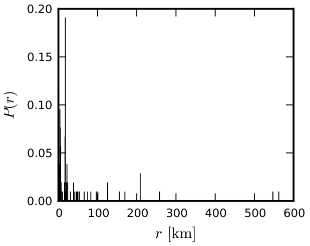	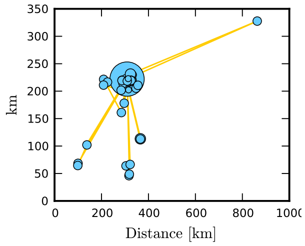	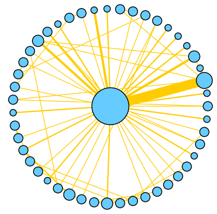
ID: 121<br>Status: Employed<br>Sex: Female<br>Age: 54	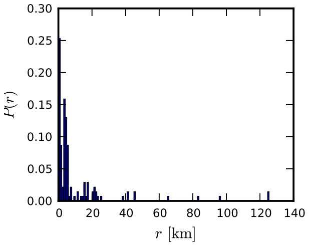	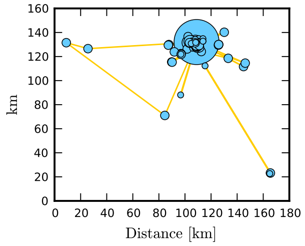	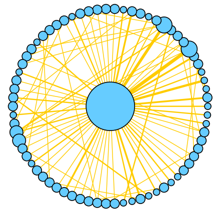
ID: 122<br>Status: Retired<br>Sex: Female<br>Age: 65	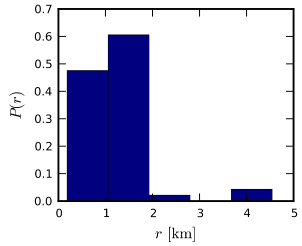	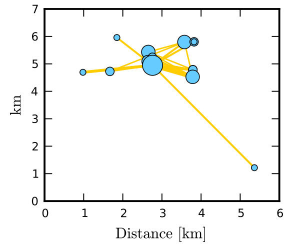	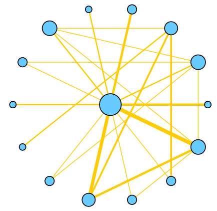
ID: 123<br>Status: Retired<br>Sex: Male<br>Age: 66	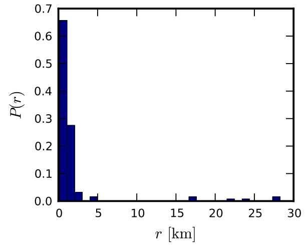	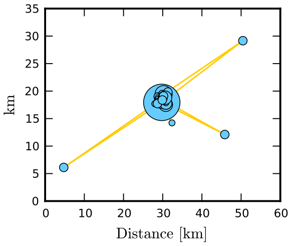	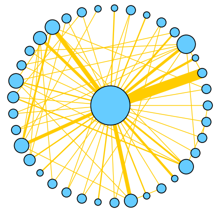
ID: 124<br>Status: Employed<br>Sex: Female<br>Age: 28	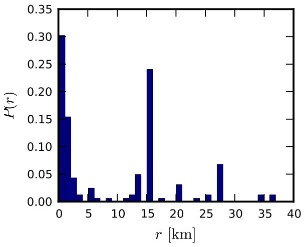	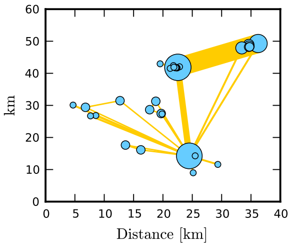	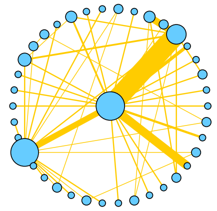

TABLE S1 (continued)

Individual ID	Displacement distribution	Mobility network (with geolocation)	Mobility network (without geolocation)
ID: 125<br>Status: Employed<br>Sex: Female<br>Age: 28	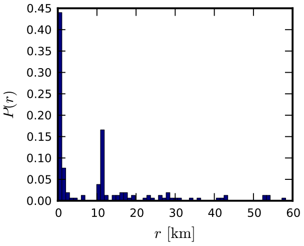	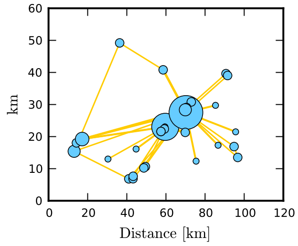	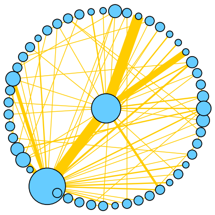
ID: 126<br>Status: Employed<br>Sex: Male<br>Age: 32	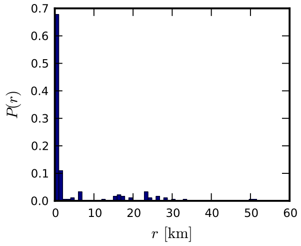	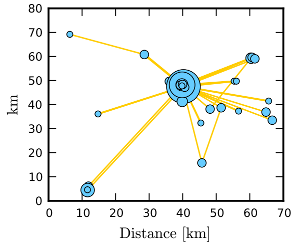	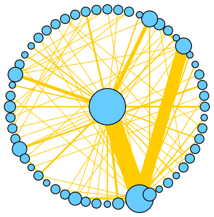
ID: 127<br>Status: Employed<br>Sex: Female<br>Age: 54	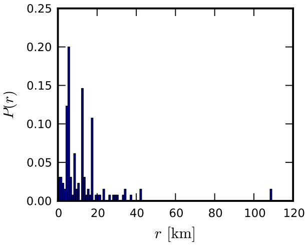	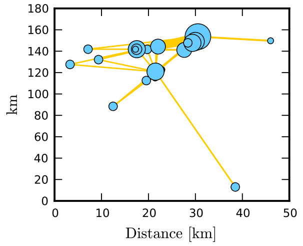	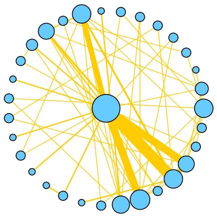
ID: 128<br>Status: Employed<br>Sex: Male<br>Age: 57	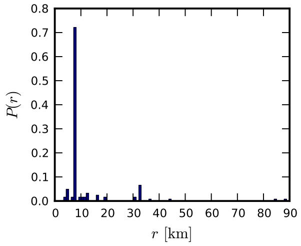	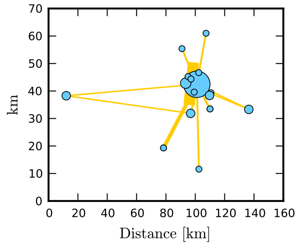	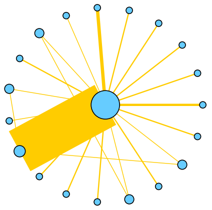
ID: 129<br>Status: Housemaker<br>Sex: Female<br>Age: 55	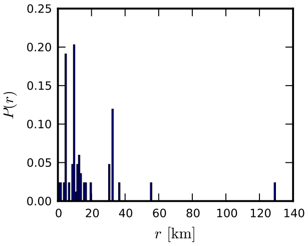	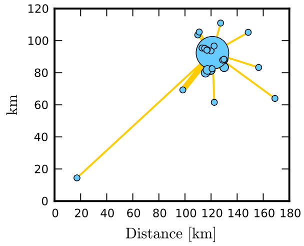	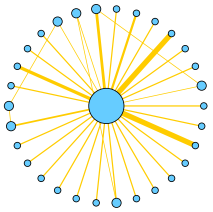
ID: 130<br>Status: Pupil<br>Sex: Female<br>Age: 14	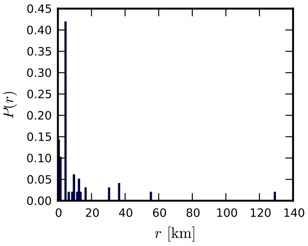	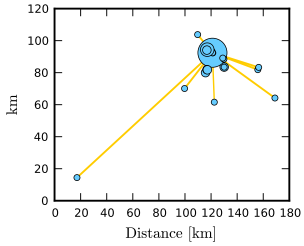	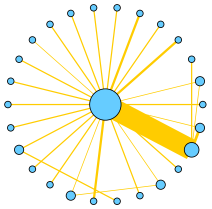

TABLE S1 (continued)

Individual ID	Displacement distribution	Mobility network (with geolocation)	Mobility network (without geolocation)
ID: 131<br>Status: Housemaker<br>Sex: Female<br>Age: 37	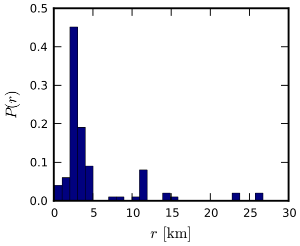	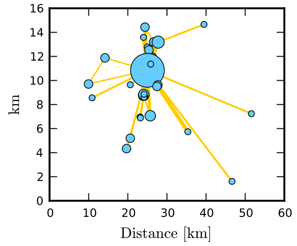	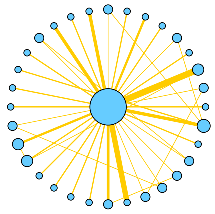
ID: 132<br>Status: Employed<br>Sex: Male<br>Age: 36	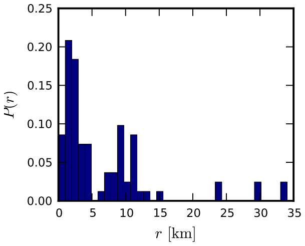	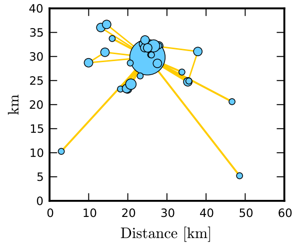	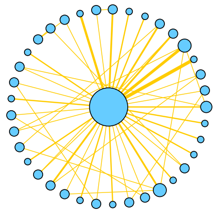
ID: 133<br>Status: Employed<br>Sex: Female<br>Age: 27	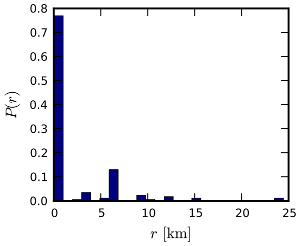	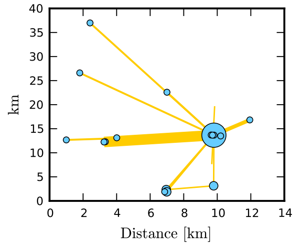	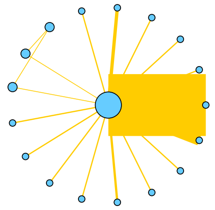
ID: 134<br>Status: Housemaker<br>Sex: Female<br>Age: 53	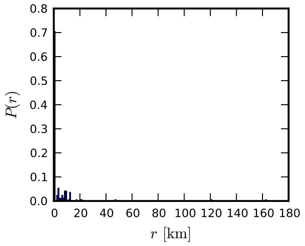	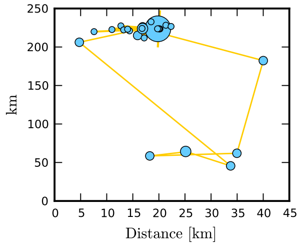	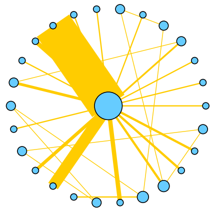
ID: 135<br>Status: Employed<br>Sex: Male<br>Age: 58	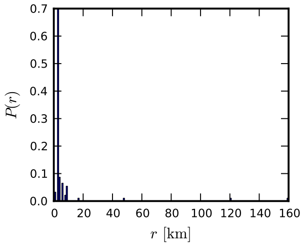	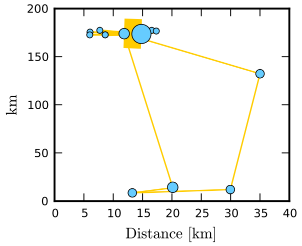	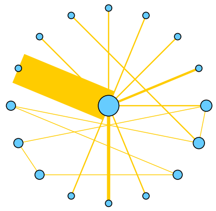
ID: 136<br>Status: Employed<br>Sex: Male<br>Age: 34	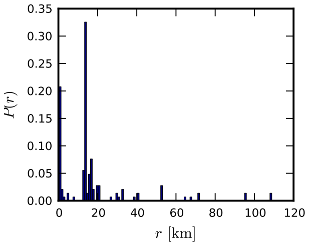	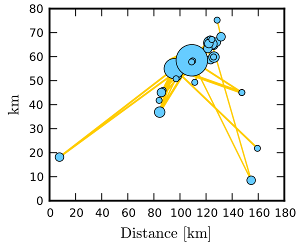	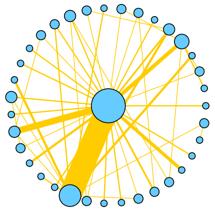

TABLE S1 (continued)

Individual ID	Displacement distribution	Mobility network (with geolocation)	Mobility network (without geolocation)
ID: 137<br>Status: Housemaker<br>Sex: Female<br>Age: 55	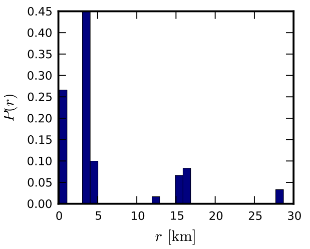	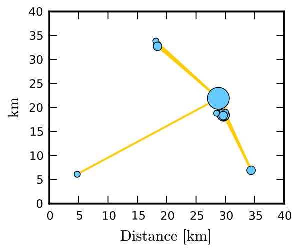	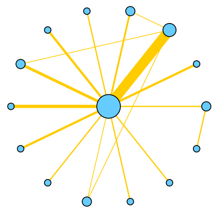
ID: 138<br>Status: Employed<br>Sex: Male<br>Age: 59	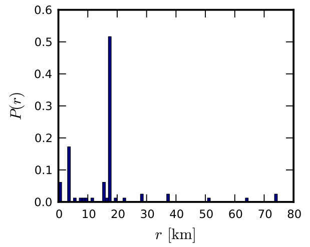	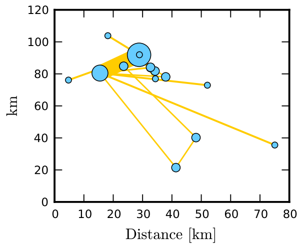	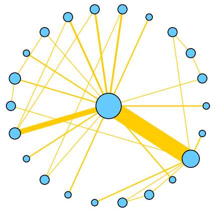
ID: 139<br>Status: Retired<br>Sex: Male<br>Age: 70	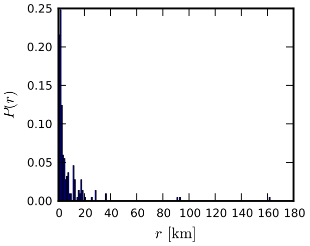	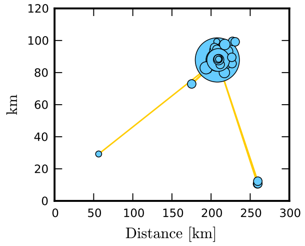	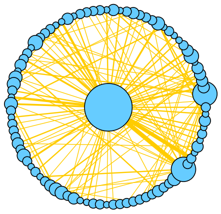
ID: 140<br>Status: Retired<br>Sex: Female<br>Age: 69	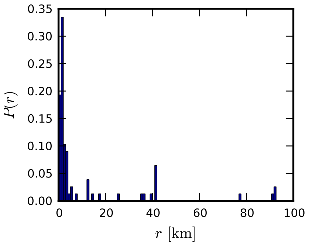	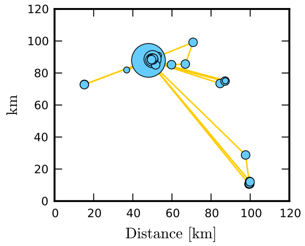	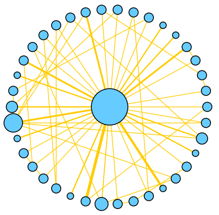
ID: 141<br>Status: Employed<br>Sex: Female<br>Age: 52	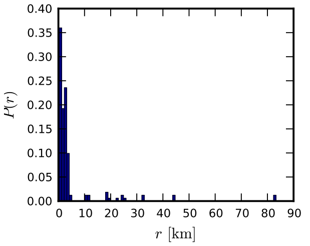	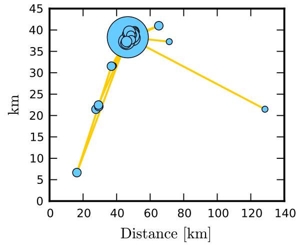	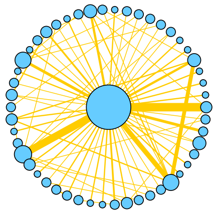
ID: 142<br>Status: Employed<br>Sex: Male<br>Age: 57	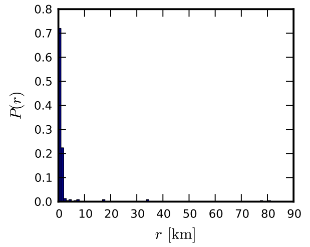	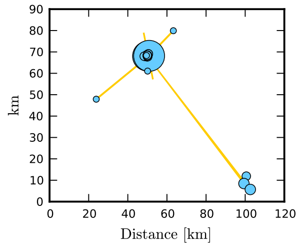	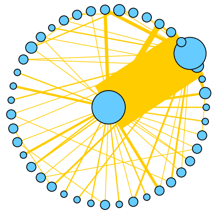

TABLE S1 (continued)

Individual ID	Displacement distribution	Mobility network (with geolocation)	Mobility network (without geolocation)
ID: 143<br>Status: Employed<br>Sex: Female<br>Age: 52	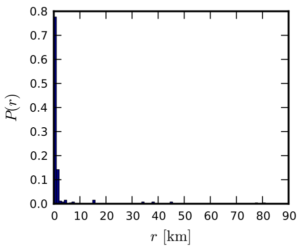	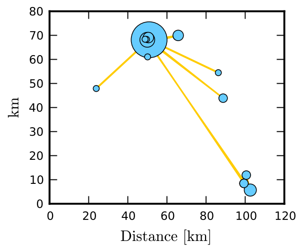	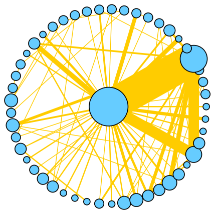
ID: 144<br>Status: Student<br>Sex: Female<br>Age: 19	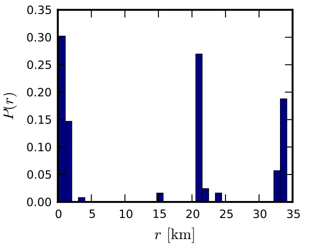	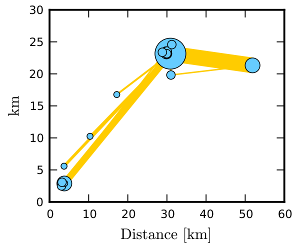	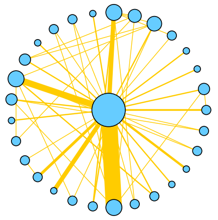
ID: 145<br>Status: Pupil<br>Sex: Male<br>Age: 17	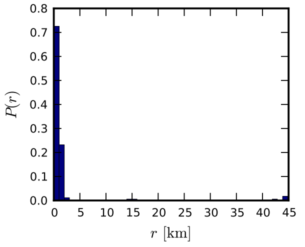	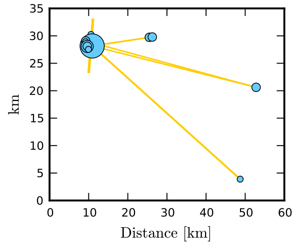	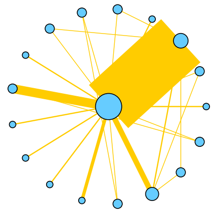
ID: 146<br>Status: Employed<br>Sex: Male<br>Age: 46	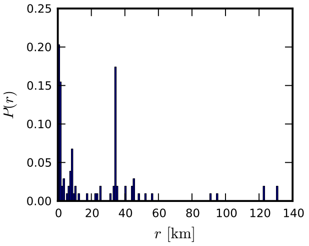	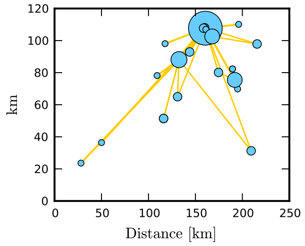	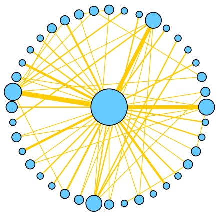
ID: 147<br>Status: Housemaker<br>Sex: Female<br>Age: 43	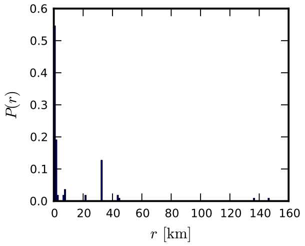	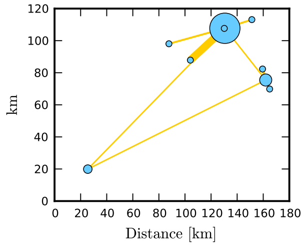	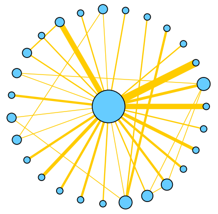
ID: 148<br>Status: Pupil<br>Sex: Male<br>Age: 11	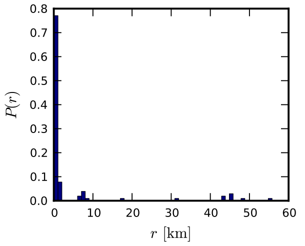	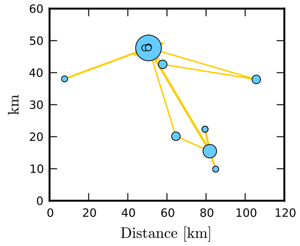	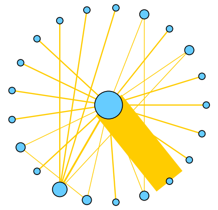

TABLE S1 (continued)

Individual ID	Displacement distribution	Mobility network (with geolocation)	Mobility network (without geolocation)
ID: 149<br>Status: Unknow<br>Sex: Female<br>Age: 30	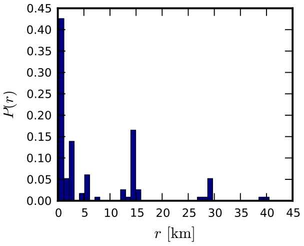	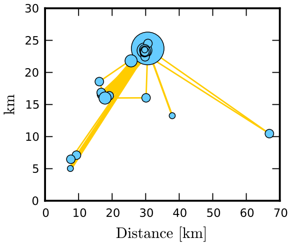	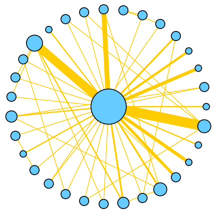
ID: 150<br>Status: Pupil<br>Sex: Female<br>Age: 13	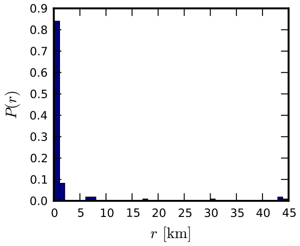	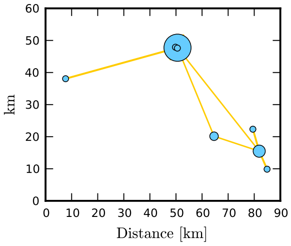	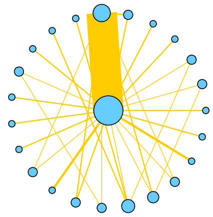
ID: 151<br>Status: Employed<br>Sex: Male<br>Age: 59	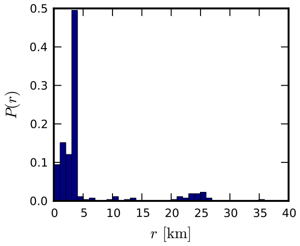	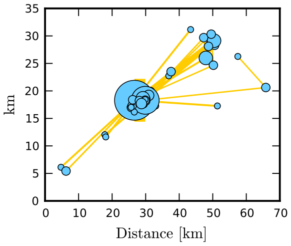	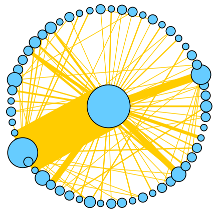
ID: 152<br>Status: Employed<br>Sex: Female<br>Age: 25	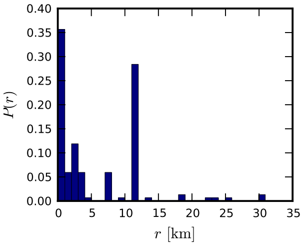	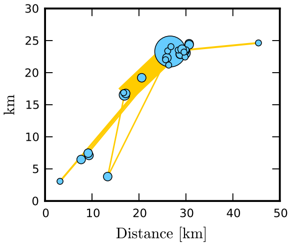	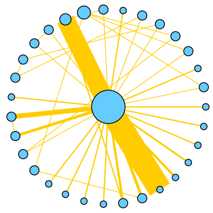
ID: 153<br>Status: Employed<br>Sex: Male<br>Age: 29	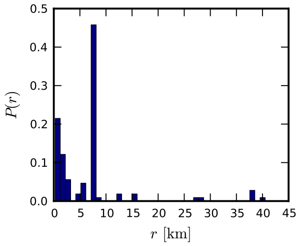	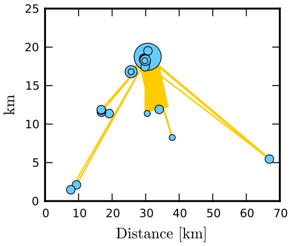	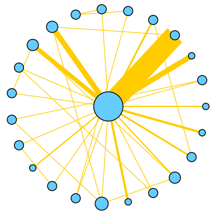
ID: 154<br>Status: Employed<br>Sex: Male<br>Age: 53	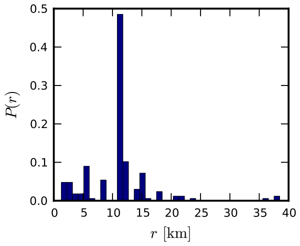	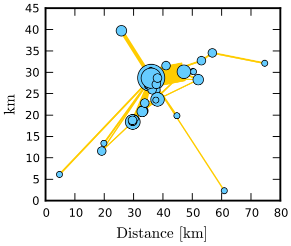	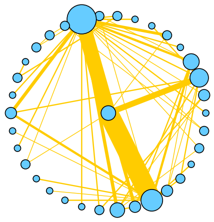

TABLE S1 (continued)

Individual ID	Displacement distribution	Mobility network (with geolocation)	Mobility network (without geolocation)
ID: 155<br>Status: Employed<br>Sex: Female<br>Age: 43	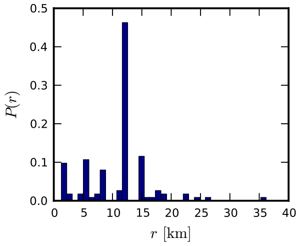	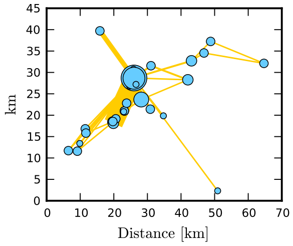	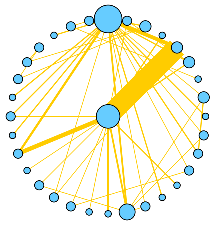
ID: 156<br>Status: Employed<br>Sex: Male<br>Age: 52	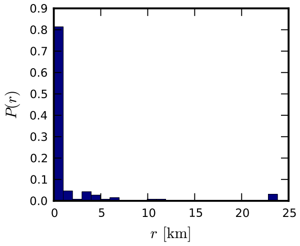	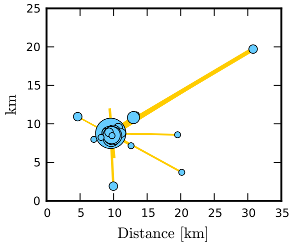	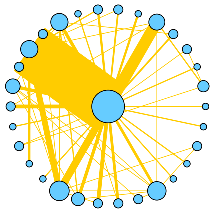
ID: 157<br>Status: Employed<br>Sex: Female<br>Age: 22	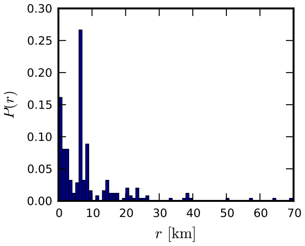	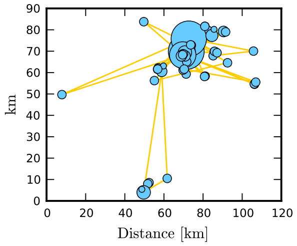	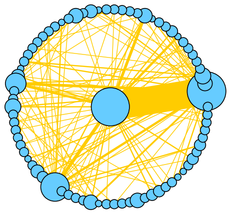
ID: 158<br>Status: Employed<br>Sex: Female<br>Age: 39	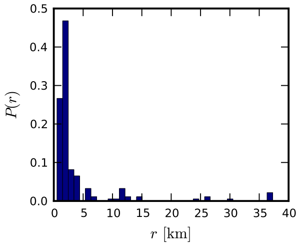	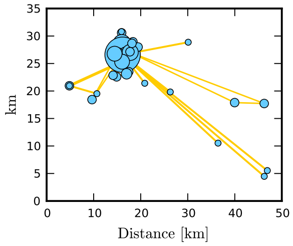	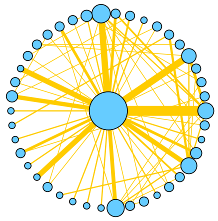
ID: 159<br>Status: Employed<br>Sex: Male<br>Age: 41	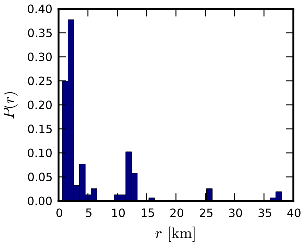	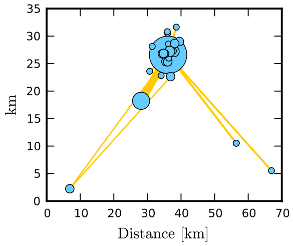	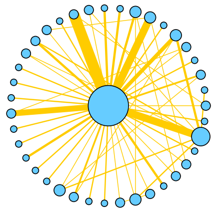
ID: 160<br>Status: Pupil<br>Sex: Male<br>Age: 13	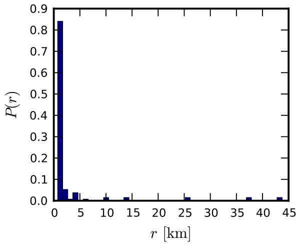	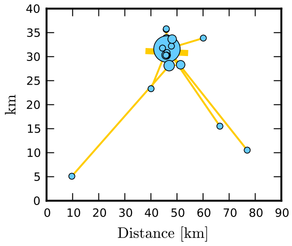	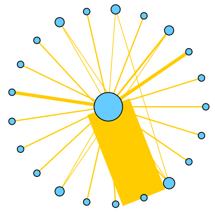

TABLE S1 (continued)

Individual ID	Displacement distribution	Mobility network (with geolocation)	Mobility network (without geolocation)
ID: 161<br>Status: Pupil<br>Sex: Female<br>Age: 15	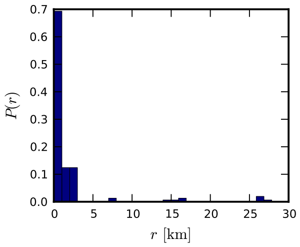	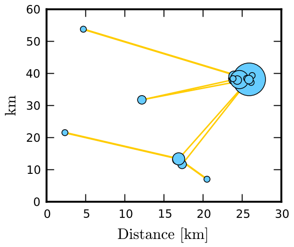	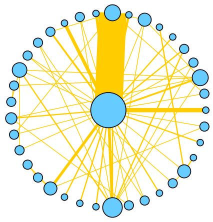
ID: 162<br>Status: Employed<br>Sex: Male<br>Age: 56	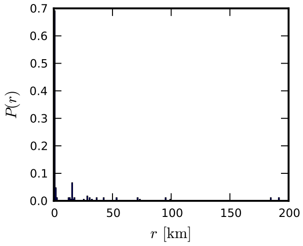	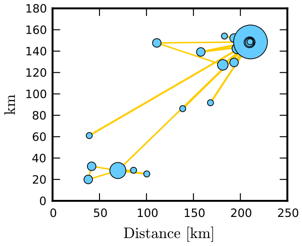	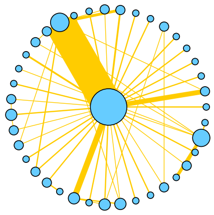
ID: 163<br>Status: Housemaker<br>Sex: Female<br>Age: 52	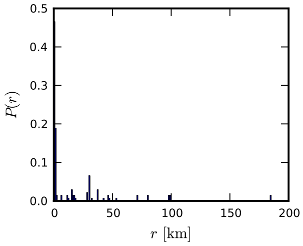	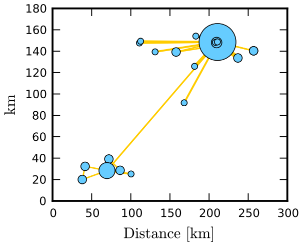	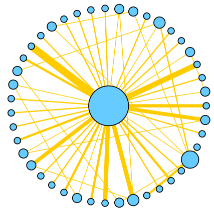
ID: 164<br>Status: Housemaker<br>Sex: Female<br>Age: 48	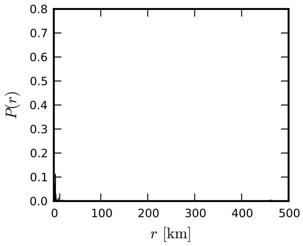	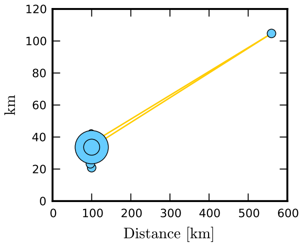	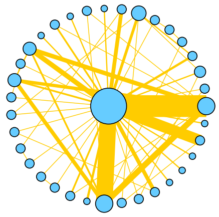
ID: 165<br>Status: Pupil<br>Sex: Male<br>Age: 12	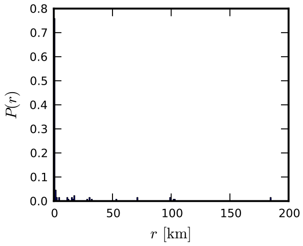	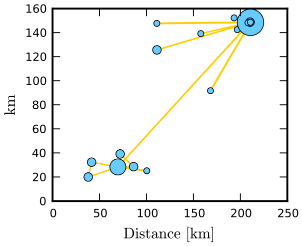	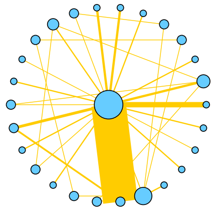
ID: 166<br>Status: Pupil<br>Sex: Male<br>Age: 10	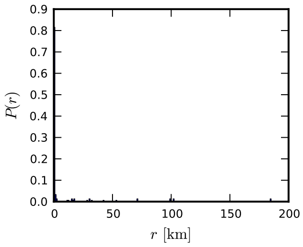	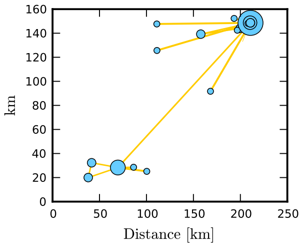	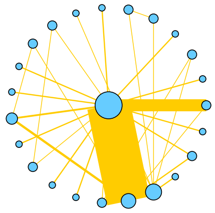

TABLE S1 (continued)

Individual ID	Displacement distribution	Mobility network (with geolocation)	Mobility network (without geolocation)
ID: 167<br>Status: Employed<br>Sex: Male<br>Age: 55	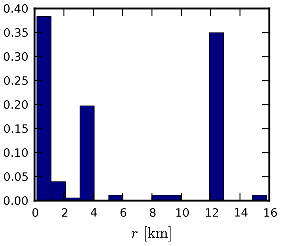	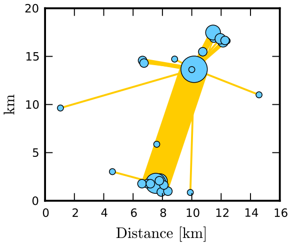	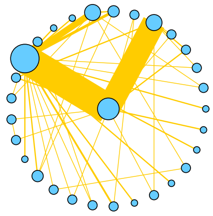
ID: 168<br>Status: Employed<br>Sex: Female<br>Age: 40	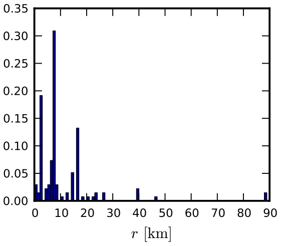	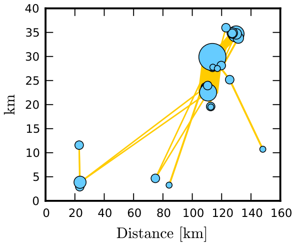	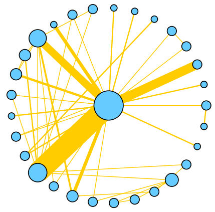
ID: 169<br>Status: Employed<br>Sex: Male<br>Age: 46	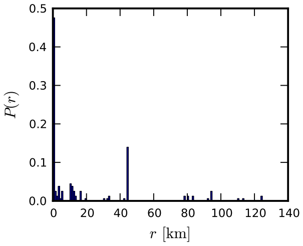	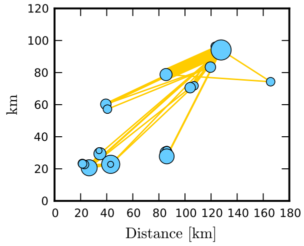	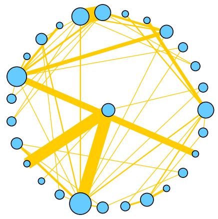
ID: 170<br>Status: Unknow<br>Sex: Male<br>Age: 46	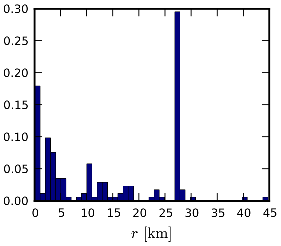	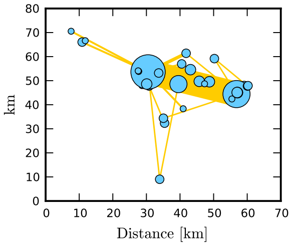	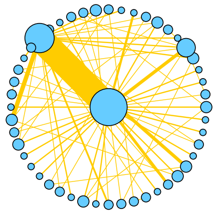
ID: 171<br>Status: Unknow<br>Sex: Female<br>Age: 41	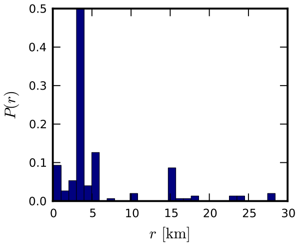	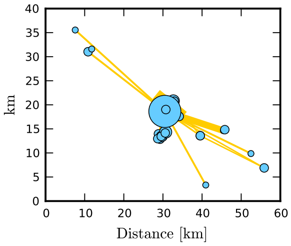	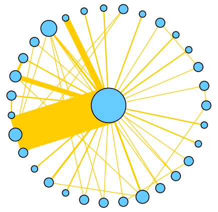
ID: 172<br>Status: Pupil<br>Sex: Female<br>Age: 15	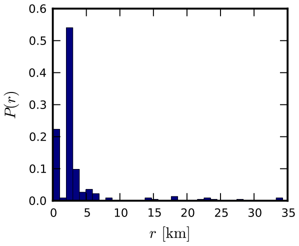	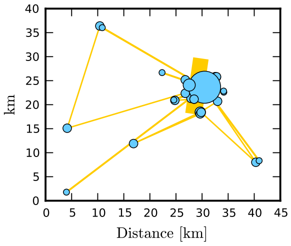	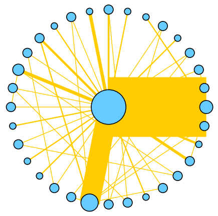

TABLE S1 (continued)

Individual ID	Displacement distribution	Mobility network (with geolocation)	Mobility network (without geolocation)
ID: 173<br>Status: Pupil<br>Sex: Male<br>Age: 15	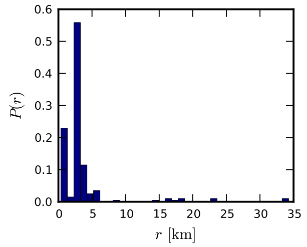	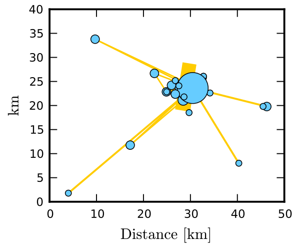	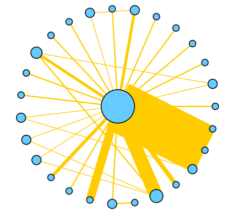
ID: 174<br>Status: Student<br>Sex: Female<br>Age: 20	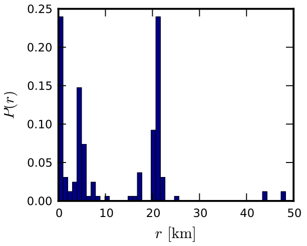	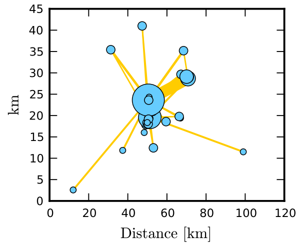	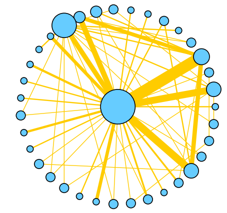
ID: 175<br>Status: Retired<br>Sex: Female<br>Age: 61	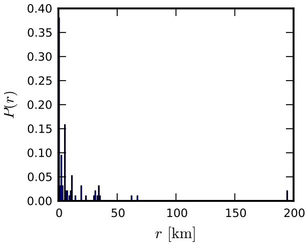	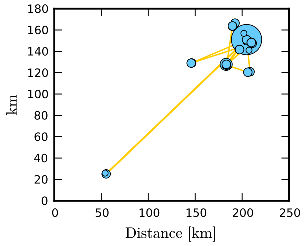	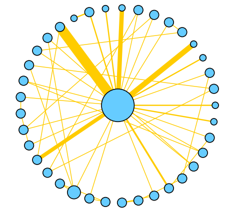
ID: 176<br>Status: Employed<br>Sex: Male<br>Age: 46	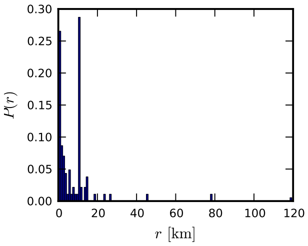	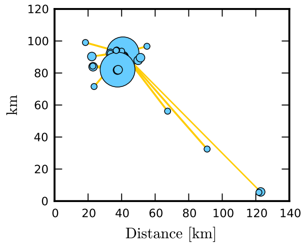	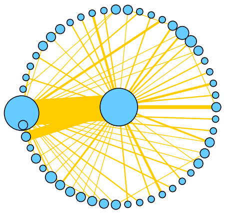
ID: 177<br>Status: Apprentice<br>Sex: Male<br>Age: 16	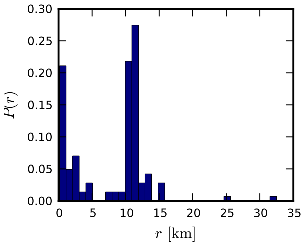	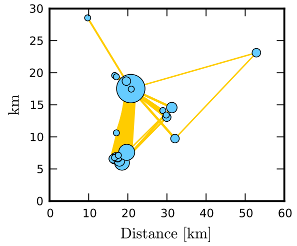	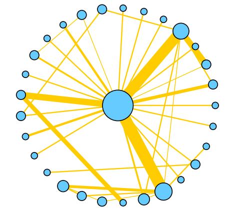
ID: 178<br>Status: Apprentice<br>Sex: Female<br>Age: 17	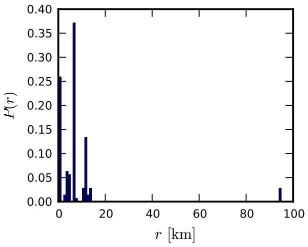	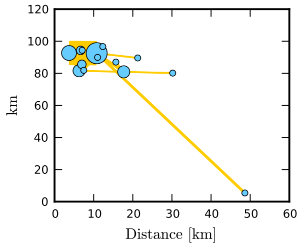	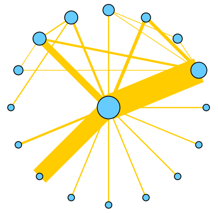

TABLE S1 (continued)

Individual ID	Displacement distribution	Mobility network (with geolocation)	Mobility network (without geolocation)
ID: 179<br>Status: Unknow<br>Sex: Female<br>Age: 40	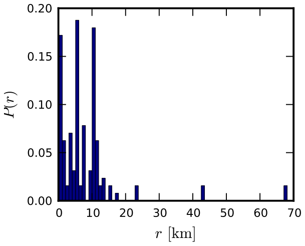	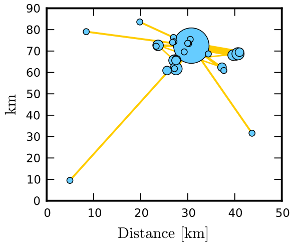	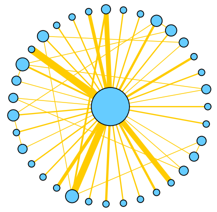
ID: 180<br>Status: Pupil<br>Sex: Female<br>Age: 14	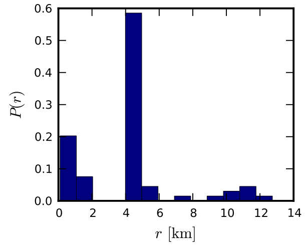	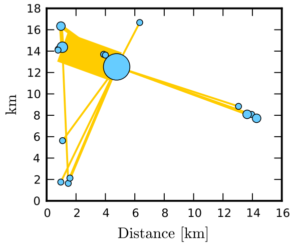	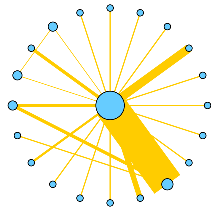
ID: 181<br>Status: Employed<br>Sex: Male<br>Age: 42	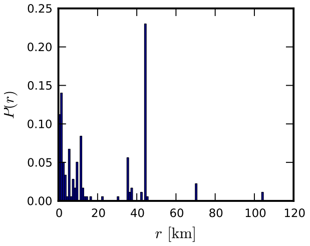	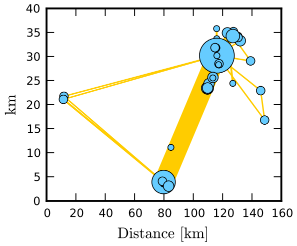	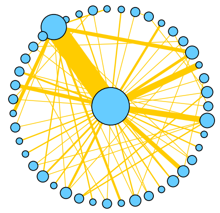
ID: 182<br>Status: Employed<br>Sex: Male<br>Age: 54	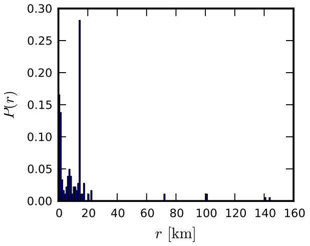	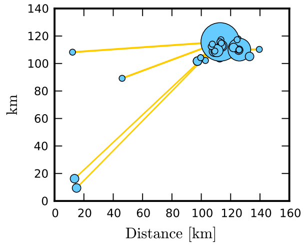	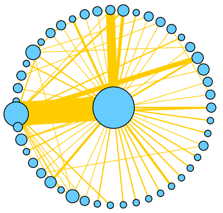
ID: 183<br>Status: Employed<br>Sex: Male<br>Age: 46	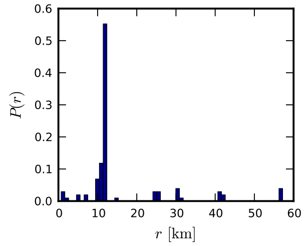	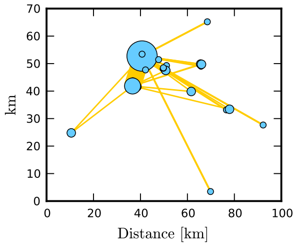	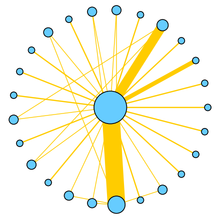
ID: 184<br>Status: Pupil<br>Sex: Male<br>Age: 14	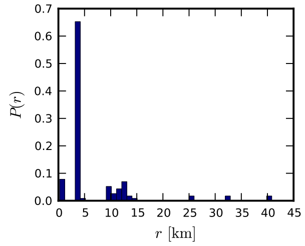	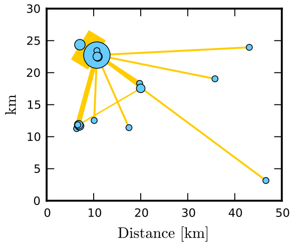	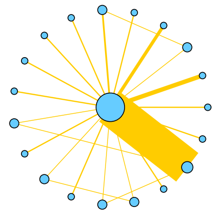

TABLE S1 (continued)

Individual ID	Displacement distribution	Mobility network (with geolocation)	Mobility network (without geolocation)
ID: 185<br>Status: Apprentice<br>Sex: Male<br>Age: 18	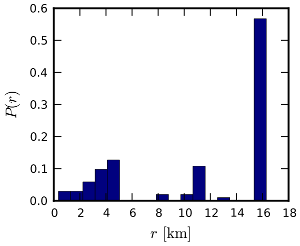	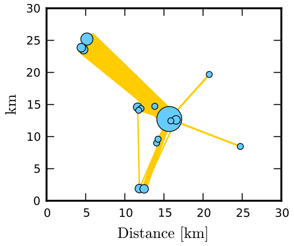	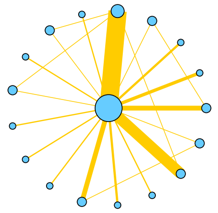
ID: 186<br>Status: Unknow<br>Sex: Female<br>Age: 44	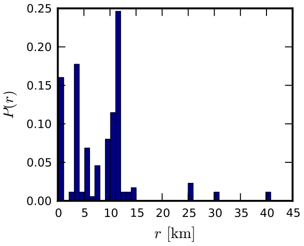	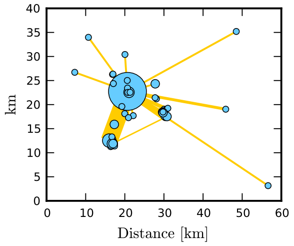	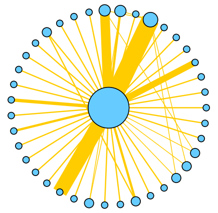
ID: 187<br>Status: Pupil<br>Sex: Female<br>Age: 16	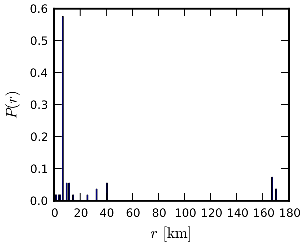	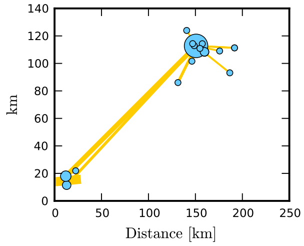	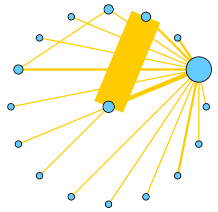
ID: 188<br>Status: Pupil<br>Sex: Male<br>Age: 14	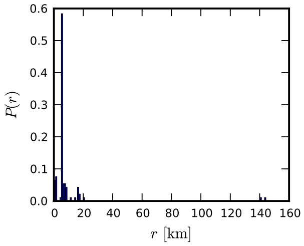	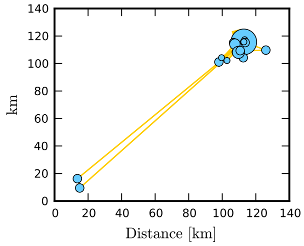	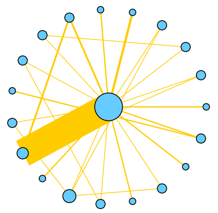
ID: 189<br>Status: Pupil<br>Sex: Female<br>Age: 12	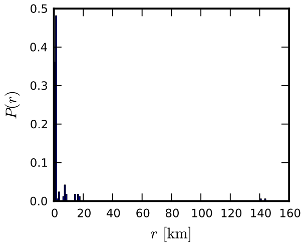	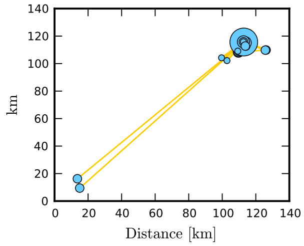	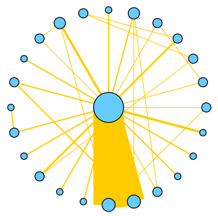
ID: 190<br>Status: Pupil<br>Sex: Female<br>Age: 10	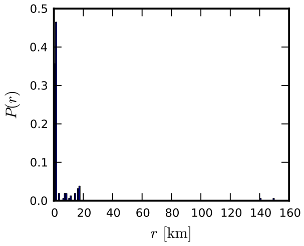	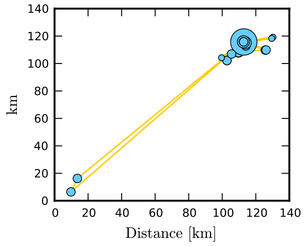	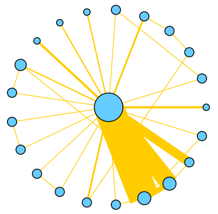

TABLE S1 (continued)

Individual ID	Displacement distribution	Mobility network (with geolocation)	Mobility network (without geolocation)
ID: 191<br>Status: Unknow<br>Sex: Male<br>Age: 51	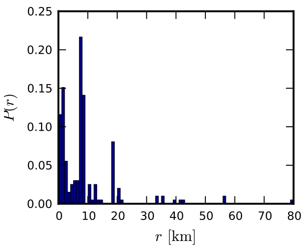	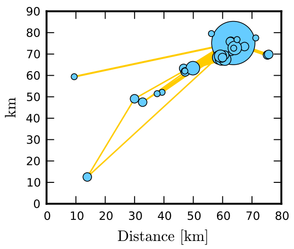	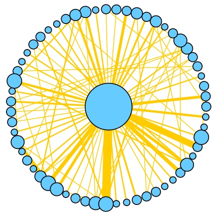
ID: 192<br>Status: Employed<br>Sex: Male<br>Age: 49	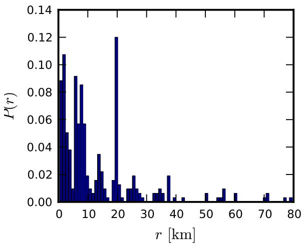	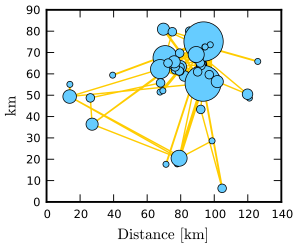	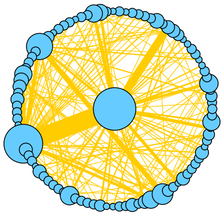
ID: 193<br>Status: Pupil<br>Sex: Male<br>Age: 10	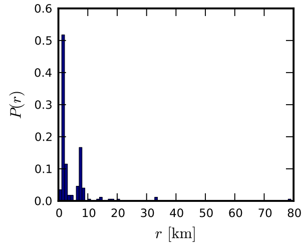	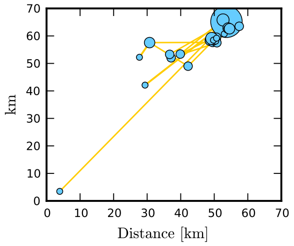	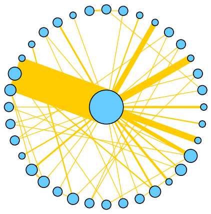
ID: 194<br>Status: Housemaker<br>Sex: Female<br>Age: 42	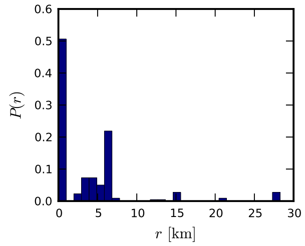	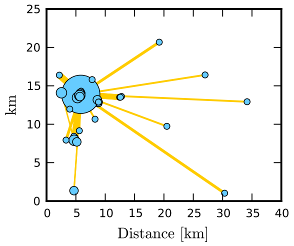	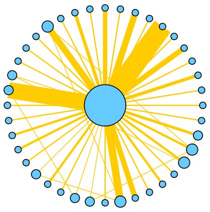
ID: 195<br>Status: Pupil<br>Sex: Female<br>Age: 13	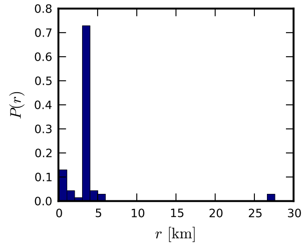	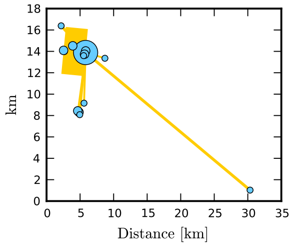	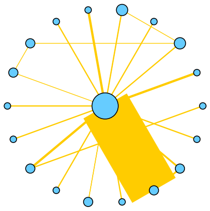
ID: 196<br>Status: Pupil<br>Sex: Female<br>Age: 10	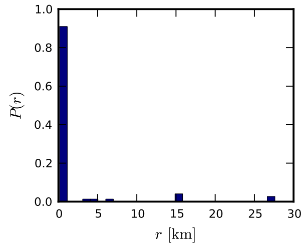	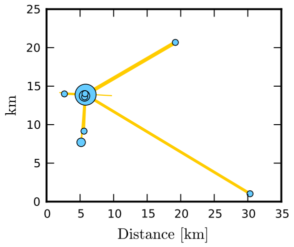	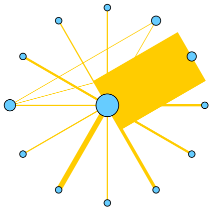

TABLE S1 (continued)

Individual ID	Displacement distribution	Mobility network (with geolocation)	Mobility network (without geolocation)
ID: 197<br>Status: Employed<br>Sex: Male<br>Age: 43	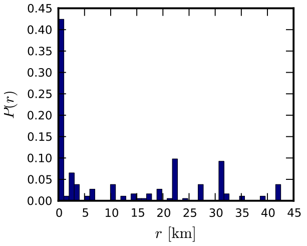	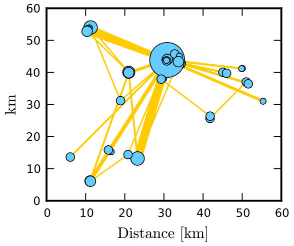	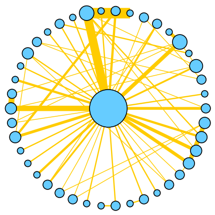
ID: 198<br>Status: Employed<br>Sex: Male<br>Age: 48	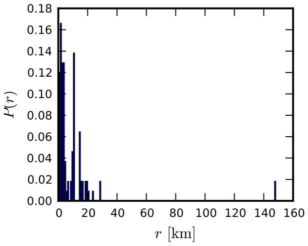	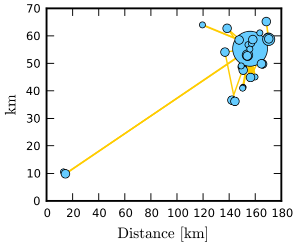	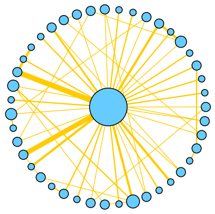
ID: 199<br>Status: Housemaker<br>Sex: Female<br>Age: 45	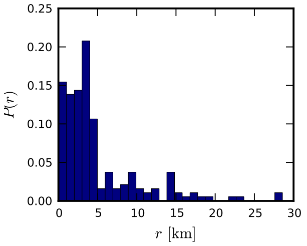	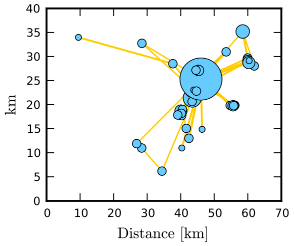	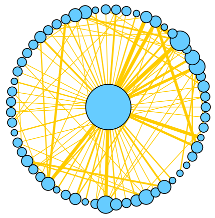
ID: 200<br>Status: Housemaker<br>Sex: Female<br>Age: 44	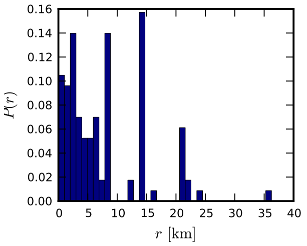	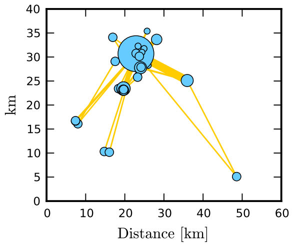	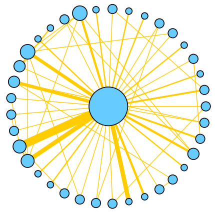
ID: 201<br>Status: Apprentice<br>Sex: Female<br>Age: 20	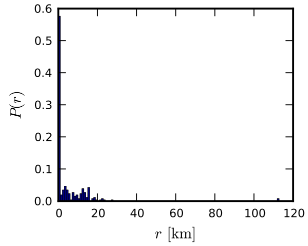	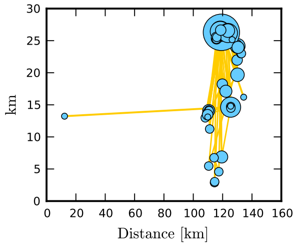	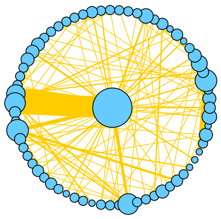
ID: 202<br>Status: Unknow<br>Sex: Female<br>Age: 68	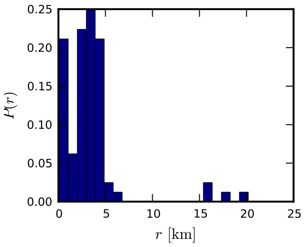	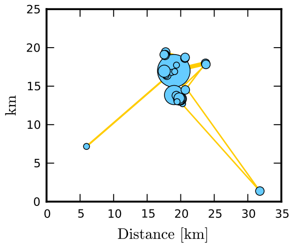	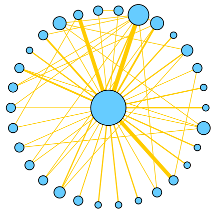

TABLE S1 (continued)

Individual ID	Displacement distribution	Mobility network (with geolocation)	Mobility network (without geolocation)
ID: 203<br>Status: Retired<br>Sex: Male<br>Age: 71	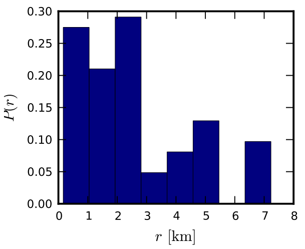	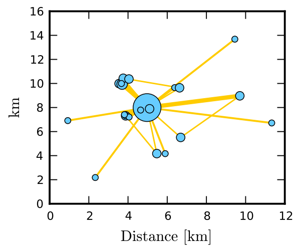	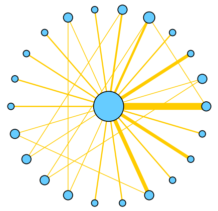
ID: 204<br>Status: Employed<br>Sex: Male<br>Age: 41	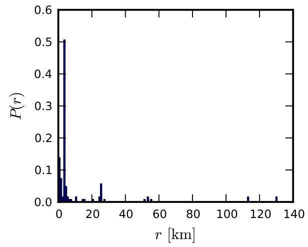	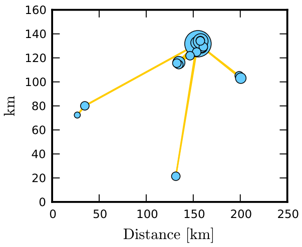	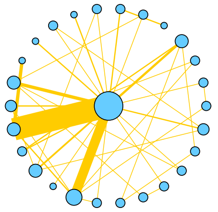
ID: 205<br>Status: Unknow<br>Sex: Female<br>Age: 47	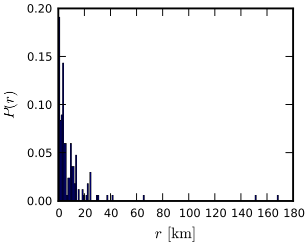	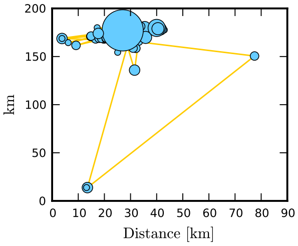	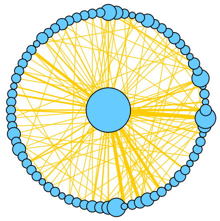
ID: 206<br>Status: Employed<br>Sex: Male<br>Age: 35	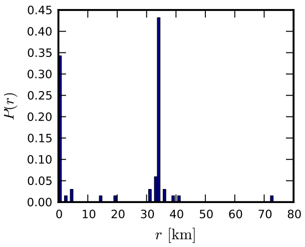	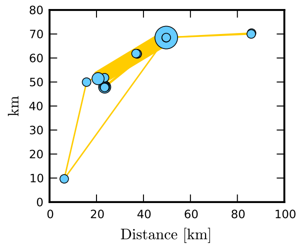	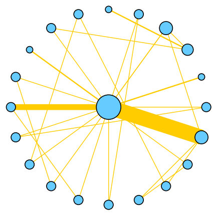
ID: 207<br>Status: Employed<br>Sex: Female<br>Age: 58	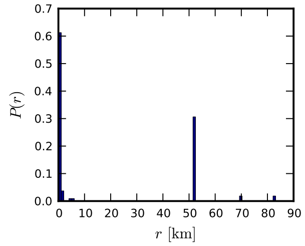	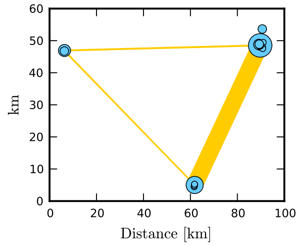	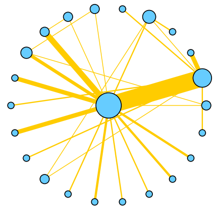
ID: 208<br>Status: Employed<br>Sex: Male<br>Age: 41	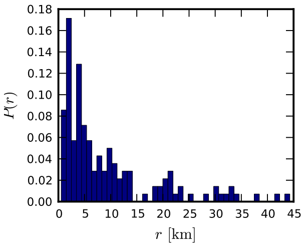	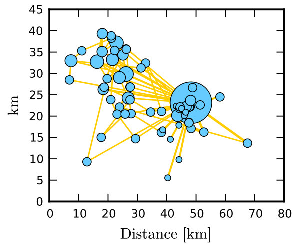	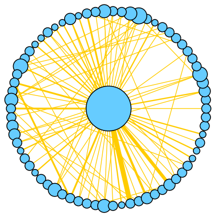

TABLE S1 (continued)

<table>
<tr><th>Individual ID</th><th>Displacement distribution</th><th>Mobility network
(with geolocation)</th><th>Mobility network
(without geolocation)</th></tr>
<tr><td>ID: 209
Status: Unknow
Sex: Female
Age: 38</td><td>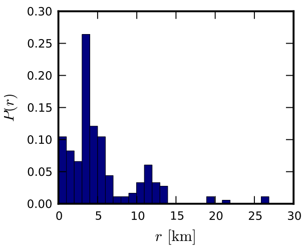
</td><td>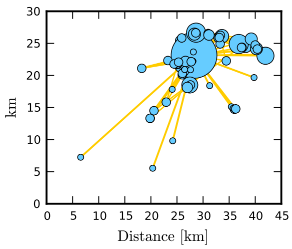
</td><td>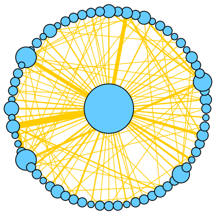</td></tr>
<tr><td>ID: 210
Status: Pupil
Sex: Male
Age: 12</td><td>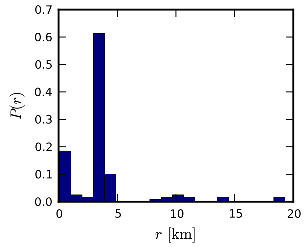
</td><td>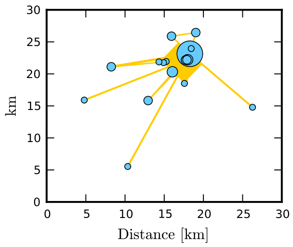
</td><td>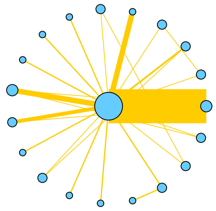</td></tr>
<tr><td>ID: 211
Status: Apprentice
Sex: Female
Age: 18</td><td>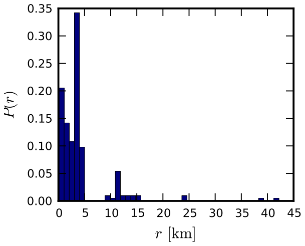
</td><td>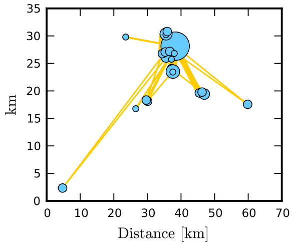
</td><td>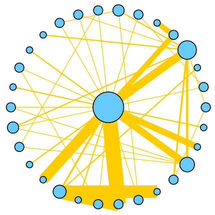</td></tr>
<tr><td>ID: 212
Status: Apprentice
Sex: Male
Age: 17</td><td>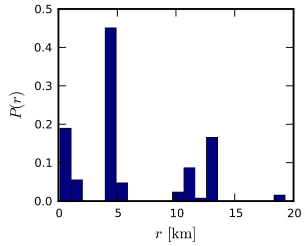
</td><td>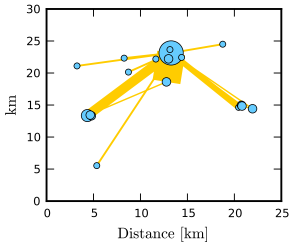
</td><td>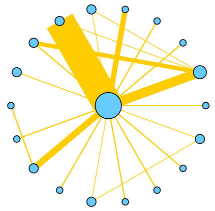</td></tr>
<tr><td>ID: 213
Status: Employed
Sex: Male
Age: 51</td><td>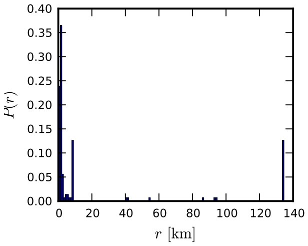
</td><td>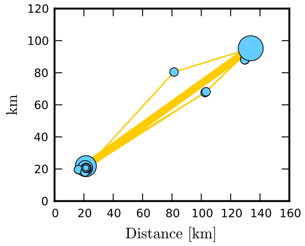
</td><td>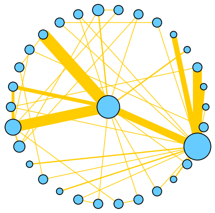</td></tr>
<tr><td>ID: 214
Status: Employed
Sex: Male
Age: 23</td><td>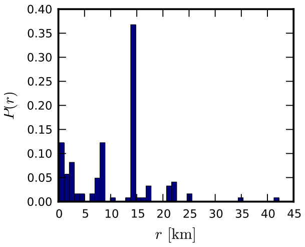
</td><td>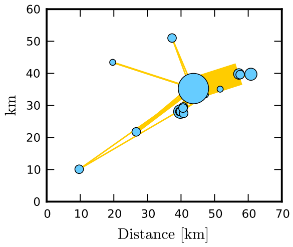
</td><td>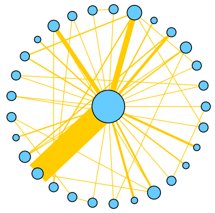</td></tr>
</table>

TABLE S1 (continued)

Individual ID	Displacement distribution	Mobility network (with geolocation)	Mobility network (without geolocation)
ID: 215<br>Status: Employed<br>Sex: Male<br>Age: 21	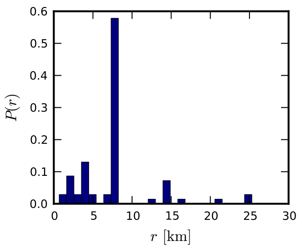	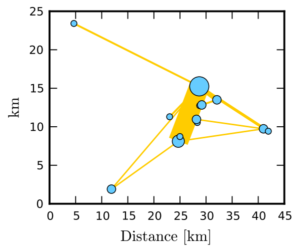	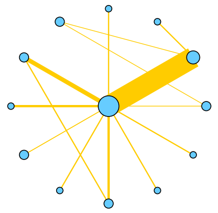
ID: 216<br>Status: Pupil<br>Sex: Male<br>Age: 15	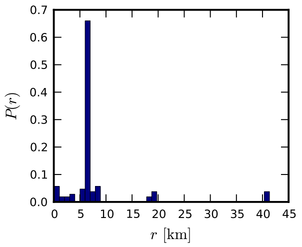	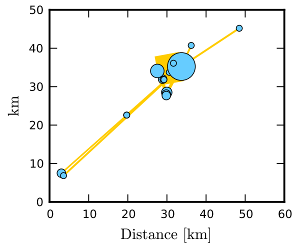	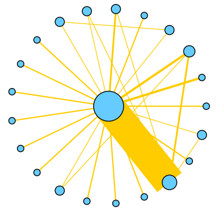
ID: 217<br>Status: Unknow<br>Sex: Female<br>Age: 49	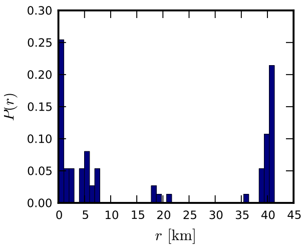	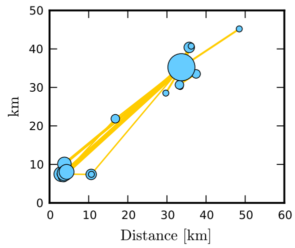	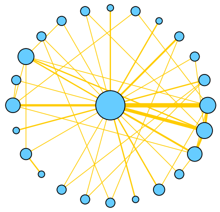
ID: 218<br>Status: Employed<br>Sex: Male<br>Age: 56	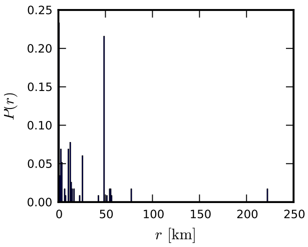	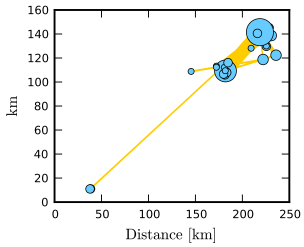	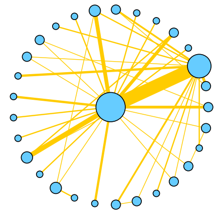
ID: 219<br>Status: Student<br>Sex: Male<br>Age: 17	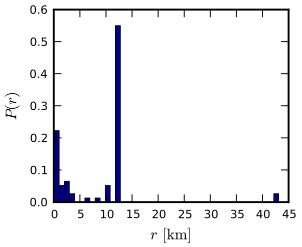	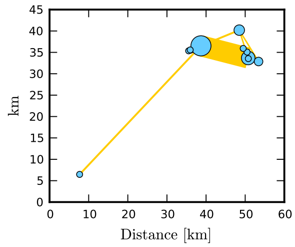	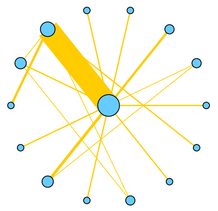
ID: 220<br>Status: Unknow<br>Sex: Female<br>Age: 44	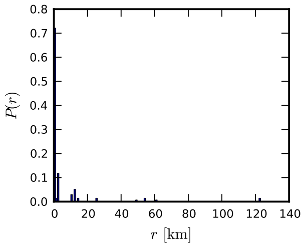	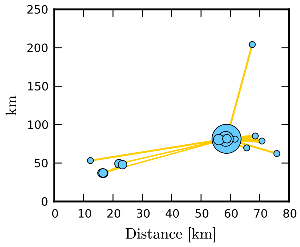	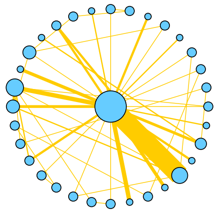

TABLE S1 (continued)

Individual ID	Displacement distribution	Mobility network (with geolocation)	Mobility network (without geolocation)
ID: 221<br>Status: Pupil<br>Sex: Male<br>Age: 14	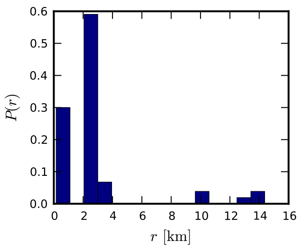	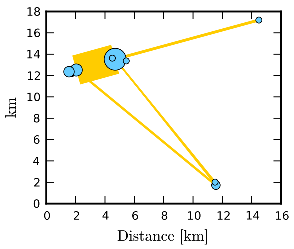	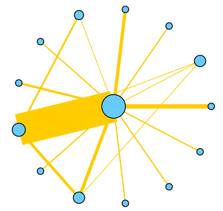
ID: 222<br>Status: Employed<br>Sex: Male<br>Age: 54	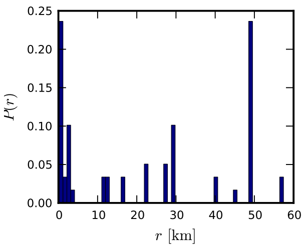	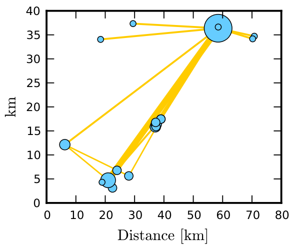	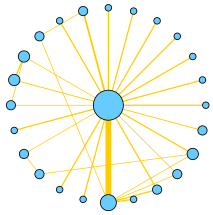
ID: 223<br>Status: Housemaker<br>Sex: Female<br>Age: 45	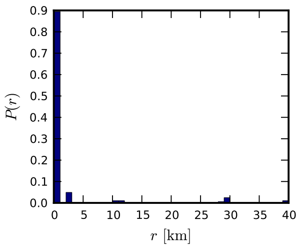	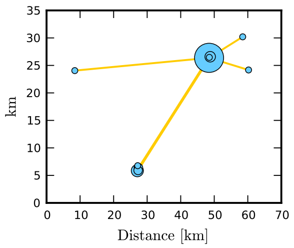	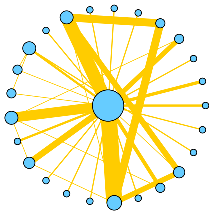
ID: 224<br>Status: Pupil<br>Sex: Female<br>Age: 12	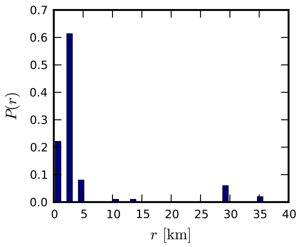	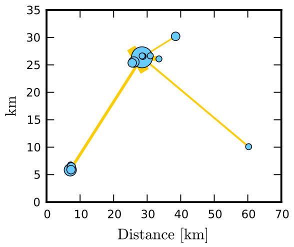	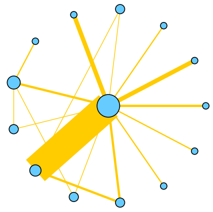
ID: 225<br>Status: Pupil<br>Sex: Female<br>Age: 10	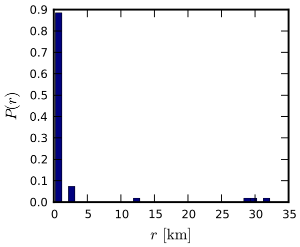	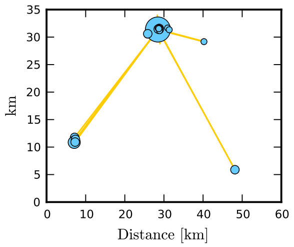	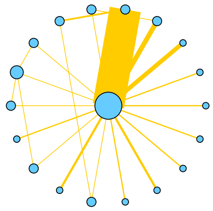
ID: 226<br>Status: Employed<br>Sex: Male<br>Age: 45	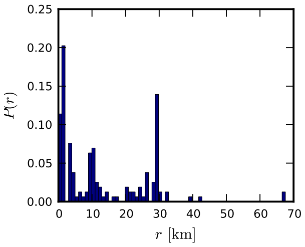	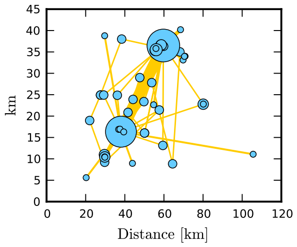	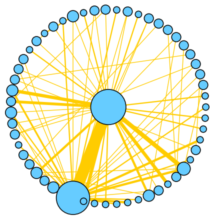

TABLE S1 (continued)

Individual ID	Displacement distribution	Mobility network (with geolocation)	Mobility network (without geolocation)
ID: 227<br>Status: Housemaker<br>Sex: Female<br>Age: 44	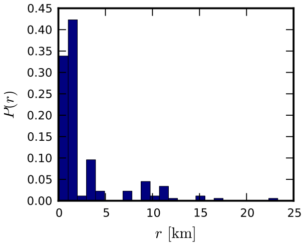	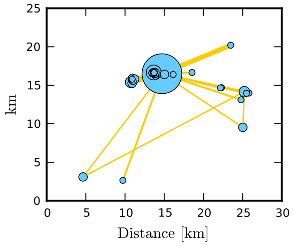	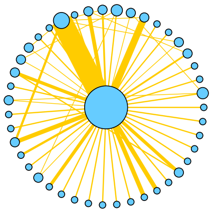
ID: 228<br>Status: Pupil<br>Sex: Female<br>Age: 12	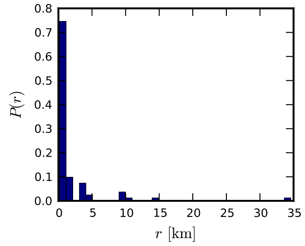	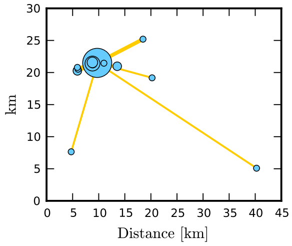	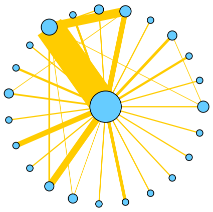
ID: 229<br>Status: Pupil<br>Sex: Male<br>Age: 17	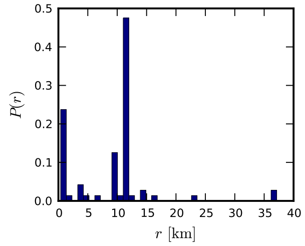	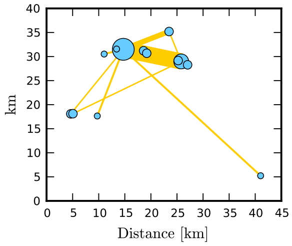	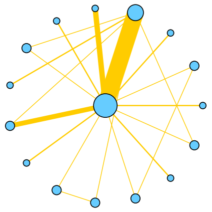
ID: 230<br>Status: Pupil<br>Sex: Female<br>Age: 15	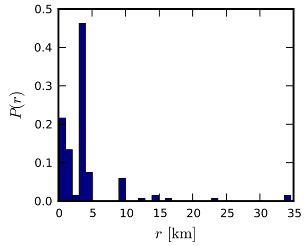	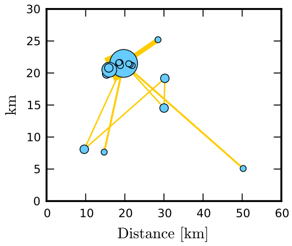	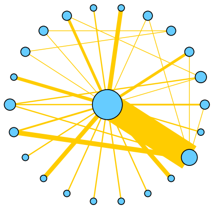

Table S2: Tests of power-law displacement distributions for all individuals by the method proposed by Clauset, Shalizi and Newman in *SIAM Rev.* 2009. For each individual, we show its ID, number of displacements (n), lower bound to the power-law behavior (x_{min}), number of displacements observed in the power-law region (n_{tail}), estimated power exponent (α), likelihood ratio for the fit to the power law (LR) and p-value for the fit to the power law (p). 202 individuals' p-value are less than 0.1 and thus the hypothesis that their displacement distributions are power-law should be ruled out.

ID	n	x_{min}	n_{tail}	α	LR	p
1	149	0.807	111	1.892	-224.328	0
2	217	0.118	217	1.269	-844.284	0
3	241	0.656	214	1.886	-391.122	0
4	103	0.993	32	1.722	-86.564	0.111
5	156	0.993	70	1.641	-209.945	0
6	220	0.301	198	2.098	-122.193	0
7	179	8.465	103	2.649	-333.988	0

TABLE S2 (continued)

ID	n	x_{min}	n_{tail}	α	LR	p
8	158	1.917	129	1.617	-484.385	0
9	104	0.393	102	1.302	-466.731	0
10	148	0.475	48	1.574	-122.57	0.075
11	139	2.457	112	1.579	-467.371	0
12	83	2.023	81	1.568	-326.533	0
13	80	4.394	64	2.569	-170.665	0.198
14	84	7.174	12	11.462	-8.62	0
15	123	5.82	106	2.336	-341.359	0
16	183	0.514	153	1.83	-264.13	0
17	84	0.571	74	1.564	-206.086	0
18	153	0.696	133	1.664	-339.642	0.005
19	162	0.013	162	1.193	-561.739	0
20	154	0.107	153	1.48	-242.628	0
21	126	9.417	78	2.065	-321.233	0
22	147	2.533	47	1.879	-150.203	0.002
23	235	3.156	140	2.218	-388.206	0
24	235	0.097	216	1.36	-534.352	0
25	146	0.2	146	1.416	-389.584	0
26	176	0.396	163	1.502	-449.2	0
27	126	7.032	84	1.893	-351.422	0
28	322	0.401	164	1.772	-268.75	0
29	78	8.197	34	2.544	-112.796	0.229
30	94	0.288	94	1.342	-352.574	0
31	70	8.197	32	2.473	-108.661	0.196
32	127	4.658	94	2.002	-332.217	0
33	177	1.974	52	1.595	-201.749	0
34	149	2.014	45	1.62	-170.552	0
35	179	2.353	152	2.524	-317.719	0
36	223	0.849	99	1.728	-250.365	0
37	103	0.14	103	1.575	-136.346	0
38	175	3.392	87	2.867	-185.547	0.076
39	113	0.114	112	1.303	-372.037	0
40	172	0.234	172	1.388	-528.362	0
41	285	1.988	140	2.634	-253.15	0
42	275	0.53	208	1.71	-440.309	0
43	319	1.235	140	2.109	-281.291	0
44	141	1.168	140	2.958	-139.104	0.116
45	96	0.575	91	1.649	-220.062	0
46	226	1.13	125	2.304	-202.938	0.036
47	147	1.152	52	1.662	-159.317	0.194
48	109	0.456	109	1.313	-498.751	0
49	136	1.152	124	1.814	-319.409	0
50	106	0.93	100	1.431	-409.056	0
51	115	2.943	99	2.641	-217.15	0
52	136	1.865	42	1.63	-154.335	0.007
53	125	1.346	54	1.62	-182.933	0.038
54	89	5.807	60	1.851	-245.753	0
55	202	2.286	167	2.849	-292.81	0
56	202	2.717	98	2.202	-259.483	0
57	96	2.692	94	1.769	-333.881	0
58	89	1.417	45	1.498	-182.524	0
59	87	0.206	87	1.249	-420.526	0

TABLE S2 (continued)

ID	n	x_{min}	n_{tail}	α	LR	p
60	134	8.12	107	2.323	-382.011	0
61	97	1.785	19	1.544	-76.519	0.420
62	89	0.047	89	1.189	-434.104	0
63	162	0.644	151	2.454	-131.757	0
64	239	1.711	80	1.963	-209.128	0.231
65	160	0.702	62	1.966	-106.386	0
66	149	3.337	23	1.781	-85.844	0.835
67	106	0.887	101	1.616	-301.644	0
68	140	1.98	52	1.722	-176.461	0
69	171	1.962	65	1.66	-234.237	0.001
70	190	0.18	190	1.309	-703.822	0
71	179	8.563	102	2.921	-307.523	0.020
72	84	1.346	72	1.87	-186.244	0
73	82	1.4	66	1.819	-181.962	0
74	229	0.269	227	2.587	32.735	0
75	111	2.898	110	2.615	-242.417	0
76	253	0.221	247	1.481	-568.343	0
77	206	0.292	206	1.403	-651.177	0
78	174	0.223	174	1.6	-292.147	0
79	187	0.093	187	1.292	-612.454	0
80	153	0.716	147	1.866	-288.772	0
81	165	2.329	121	2.313	-282.498	0
82	139	0.202	133	2.243	1.67	0
83	138	0.166	136	1.851	-73.857	0
84	142	1.115	44	2.328	-69.404	0.147
85	86	1.034	83	1.897	-187.263	0
86	62	1.21	59	2.156	-112.711	0
87	111	1.164	106	1.964	-235.807	0
88	140	1.555	129	1.575	-481.662	0
89	120	0.546	120	1.651	-283.385	0
90	105	1.453	36	1.672	-117.391	0
91	224	0.175	224	1.323	-781.679	0
92	153	0.251	140	1.604	-248.967	0
93	120	0.732	114	2.082	-174.783	0
94	243	0.236	243	1.491	-559.569	0
95	127	0.174	127	1.33	-429.778	0
96	91	2.43	85	1.832	-278.266	0
97	82	0.468	82	1.373	-320.628	0
98	223	10.593	44	2.179	-177.921	0.720
99	208	1.261	68	1.703	-204.536	0.024
100	142	0.465	141	1.312	-648.763	0
101	281	0.828	272	1.724	-684.254	0
102	166	0.491	82	1.715	-165.783	0.001
103	163	3.243	102	2.041	-315.819	0
104	124	3.437	105	1.569	-478.359	0
105	148	0.468	148	1.539	-401.444	0
106	190	1.608	180	1.664	-610.003	0
107	168	4.882	105	2.51	-297.73	0
108	140	0.253	54	1.37	-179.733	0.002
109	131	0.082	131	1.212	-626.803	0
110	160	0.404	155	1.91	-199.38	0
111	138	0.298	134	1.343	-506.351	0

TABLE S2 (continued)

ID	n	x_{min}	n_{tail}	α	LR	p
112	129	0.282	125	1.328	-487.717	0
113	153	2.296	132	1.47	-622.015	0
114	167	3.998	147	1.993	-499.673	0
115	143	0.486	140	1.326	-624.553	0
116	146	0.406	145	1.433	-471.056	0
117	173	1.907	29	1.875	-84.745	0.711
118	177	0.807	158	2.566	-154.008	0
119	191	8.997	123	2.283	-458.465	0
120	105	17.743	33	1.789	-177.534	0.074
121	139	14.268	29	2.433	-115.895	0.449
122	53	0.185	53	1.656	-66.755	0
123	127	0.938	76	2.775	-70.322	0.209
124	163	0.129	163	1.304	-558.206	0
125	159	9.936	71	2.753	-234.674	0.322
126	183	0.292	161	1.626	-295.128	0
127	131	4.525	118	2.184	-375.849	0
128	127	4.413	125	2.32	-370.508	0
129	84	8.485	60	2.431	-208.74	0.084
130	98	8.555	31	2.247	-115.551	0.374
131	101	1.945	93	2.436	-185.983	0
132	84	1.285	75	1.742	-217.241	0
133	174	3.824	42	2.528	-108.027	0.137
134	167	2.943	50	1.971	-156.886	0
135	92	1.017	91	1.74	-243.012	0
136	145	7.531	109	2.078	-422.025	0
137	61	0.114	61	1.326	-183.966	0
138	82	0.676	78	1.336	-364.318	0
139	219	1.403	164	1.829	-448.258	0
140	78	1.119	63	1.688	-185.146	0.063
141	163	1.587	102	2.123	-228.089	0
142	230	0.639	190	2.687	-118.158	0
143	263	1.36	27	1.531	-103.314	0.937
144	126	0.16	126	1.278	-508.537	0
145	173	0.586	169	3.043	-40.585	0.033
146	104	0.586	99	1.375	-406.922	0
147	110	0.432	107	1.638	-233.129	0
148	103	0.591	94	2.05	-129.455	0
149	117	0.593	111	1.579	-305.281	0
150	110	0.43	99	2.272	-69.387	0
151	266	2.139	201	2.441	-419.822	0
152	152	0.356	149	1.485	-409.874	0
153	109	0.704	106	1.543	-328.554	0
154	175	1.284	175	1.504	-686.309	0
155	119	1.776	116	1.587	-441.793	0
156	260	0.424	98	1.74	-175.66	0
157	249	6.346	97	2.347	-319.321	0.127
158	190	2.319	60	2.021	-167.969	0.006
159	159	1.102	144	1.889	-336.909	0
160	135	0.732	135	2.16	-189.395	0
161	154	0.74	70	2.069	-109.763	0.066
162	167	25.919	23	2.219	-112.172	0.362
163	138	0.491	126	1.518	-362.678	0

TABLE S2 (continued)

ID	n	x_{min}	n_{tail}	α	LR	p
164	222	4.029	25	1.951	-87.378	0.137
165	131	11.083	22	1.83	-105.513	0.278
166	149	11.861	21	1.81	-103.296	0.041
167	181	0.152	181	1.364	-521.499	0
168	136	6.298	97	2.673	-283.568	0.004
169	158	0.072	158	1.305	-449.187	0
170	174	2.45	140	1.618	-559.258	0
171	154	2.45	136	2.504	-292.781	0
172	229	2.312	177	3.059	-283.474	0.045
173	207	0.376	207	1.549	-505.788	0
174	165	1.919	122	1.557	-491.767	0
175	95	5.61	34	1.879	-135.733	0.250
176	186	0.305	186	1.408	-587.387	0
177	145	0.145	145	1.289	-546.793	0
178	143	0.904	142	1.613	-428.888	0
179	130	4.069	92	2.288	-269.271	0
180	137	0.098	137	1.298	-443.824	0
181	179	1.663	158	1.502	-661.758	0
182	182	5.526	116	2.125	-403.762	0
183	104	7.157	98	2.455	-321.492	0.001
184	116	0.228	116	1.333	-420.482	0
185	109	1.51	106	1.542	-410.272	0
186	176	2.272	148	1.78	-495.862	0
187	108	6.807	40	1.612	-201.688	0.685
188	93	0.069	93	1.233	-379.879	0
189	167	6.041	22	2.297	-72.823	0.481
190	158	7.93	25	2.448	-84.775	0.450
191	199	6.37	120	2.801	-338.226	0
192	320	4.988	229	1.988	-831.541	0
193	176	0.855	172	1.9	-354.116	0
194	225	0.129	219	1.422	-479.023	0
195	141	0.107	141	1.318	-432.28	0
196	150	0.15	41	1.478	-79.244	0
197	186	0.063	186	1.274	-591.557	0
198	109	1.649	94	1.77	-287.761	0
199	191	2.826	112	2.342	-278.9	0.006
200	117	4.909	65	2.29	-202.281	0.002
201	259	0.335	230	1.556	-527.254	0
202	84	2.154	62	2.774	-108.964	0.519
203	70	1.204	53	2.225	-95.345	0.009
204	123	3.654	34	1.622	-148.917	0.017
205	168	9.229	54	2.714	-176.416	0.359
206	68	0.063	68	1.23	-276.691	0
207	109	0.109	109	1.363	-277.66	0
208	144	2.855	108	1.883	-357.145	0
209	183	3.038	137	2.591	-311.808	0.001
210	124	0.833	123	1.746	-301.323	0
211	209	2.619	136	2.936	-247.31	0.014
212	132	0.13	132	1.292	-477.117	0
213	143	2.015	63	1.484	-283.083	0
214	125	0.125	125	1.255	-526.162	0
215	73	2.463	71	1.947	-213.873	0

TABLE S2 (continued)

ID	n	x_{min}	n_{tail}	α	LR	p
216	108	3.44	98	2.347	-262.678	0
217	76	0.584	69	1.373	-284.705	0
218	116	6.904	71	1.717	-330.822	0
219	78	0.075	78	1.257	-285.52	0
220	137	0.075	137	1.486	-163.453	0
221	109	0.159	109	1.425	-257.713	0
222	60	0.185	60	1.271	-258.767	0
223	204	0.084	204	1.691	-70.44	0
224	101	0.232	101	1.438	-267.286	0
225	112	0.232	112	2.21	-19.559	0
226	159	7.275	88	2.035	-344.681	0
227	183	0.66	167	2.007	-262.051	0
228	165	0.166	165	1.539	-276.144	0
229	74	0.382	74	1.367	-278.325	0
230	136	0.291	135	1.455	-371.506	0

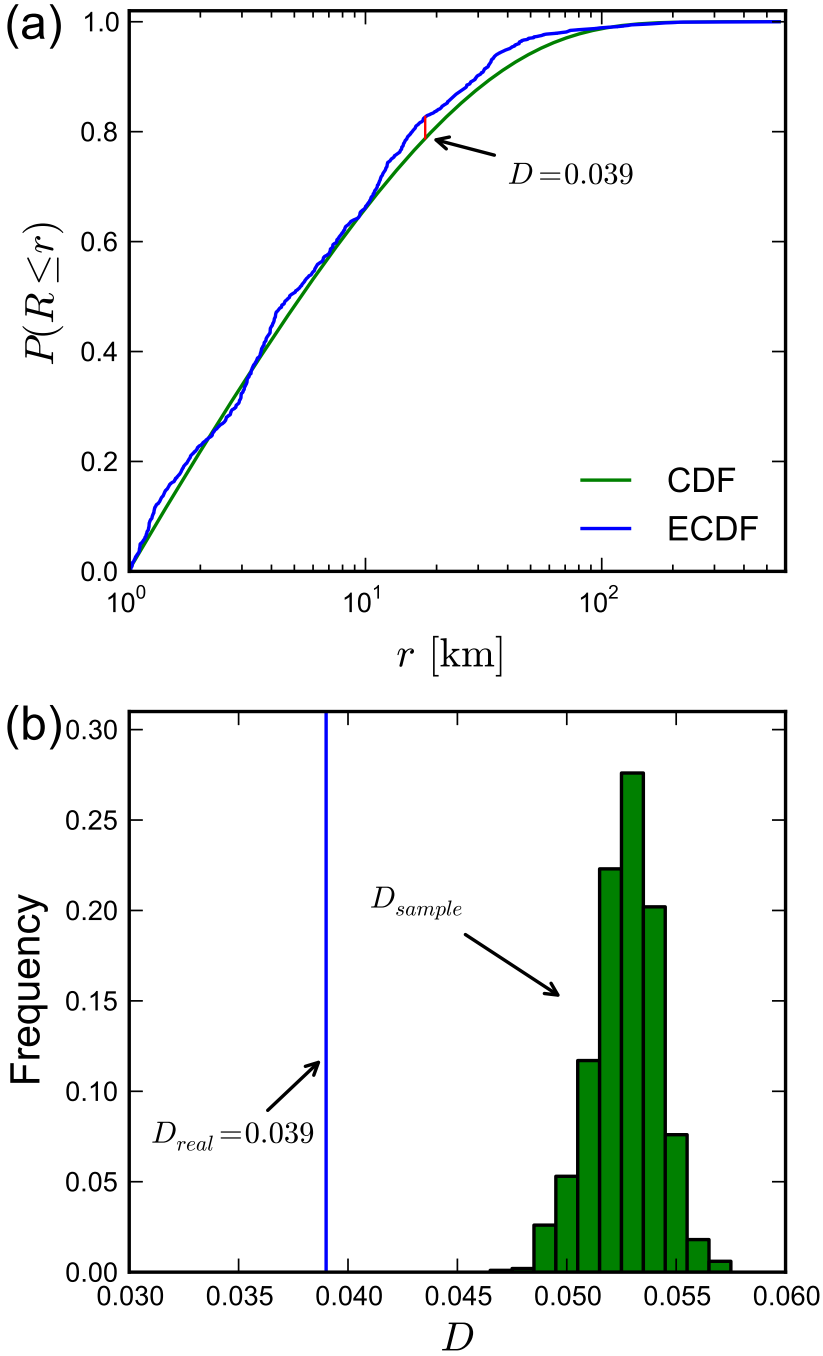

Figure S1: Kolmogorov-Smirnov test results. **a**. Illustration of the standard KS distance. Green line is the cumulative density function (CDF) of the fitting function, blue line is the empirical cumulative density function (ECDF) of the observed data, and the red line is the standard KS distance (the maximum gap between CDF and ECDF). **b**. KS distance distribution of the sampled data sets. The data sets are independently sampled from the fitting function for 1000 times. In all cases the KS distances of the sampled data (D_{sample}) are larger than the KS distance of the real data (D_{real}).

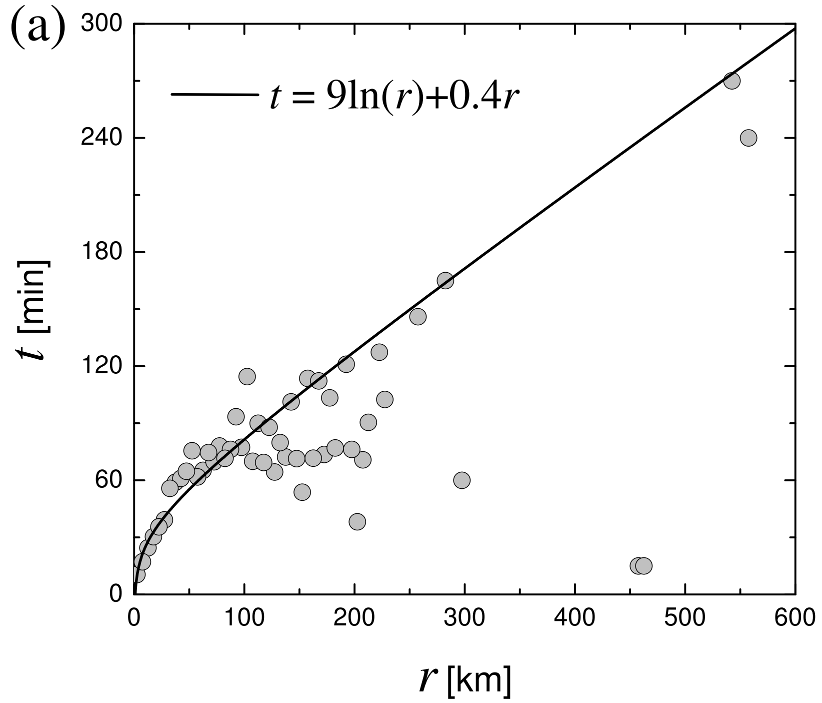

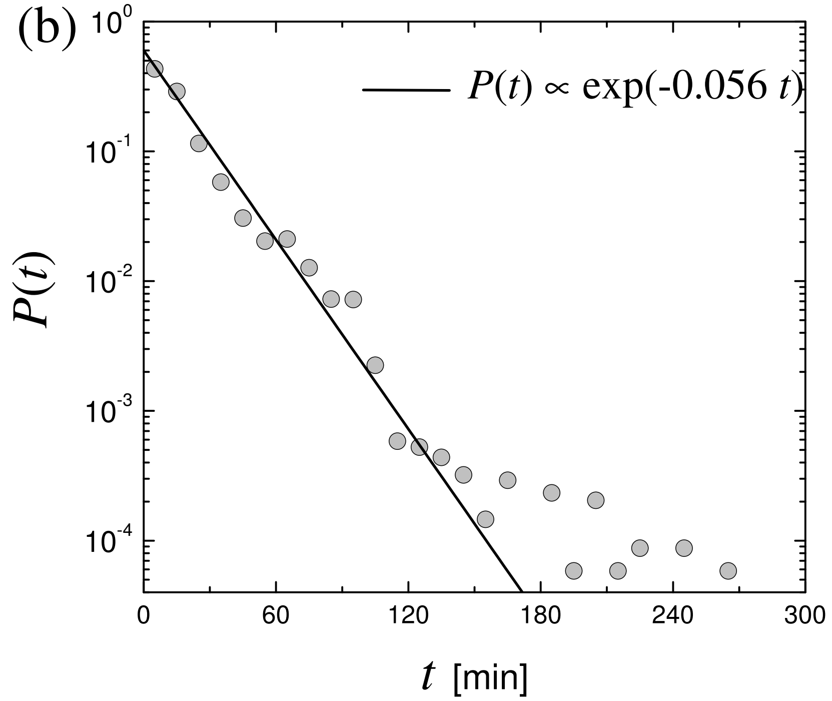

Figure S2: Statistical features of travel time in the aggregated data. **a**. The relationship between travel time t and distance r, which can be approximately fitted by a hybrid form consisting of a logarithmic term and a linear term. A few data points are not believable, for example, it is not possible to travel along more than 450 kilometers within 15 minutes. **b**. Travel time distribution at the aggregated level, which decays exponentially in the first half.

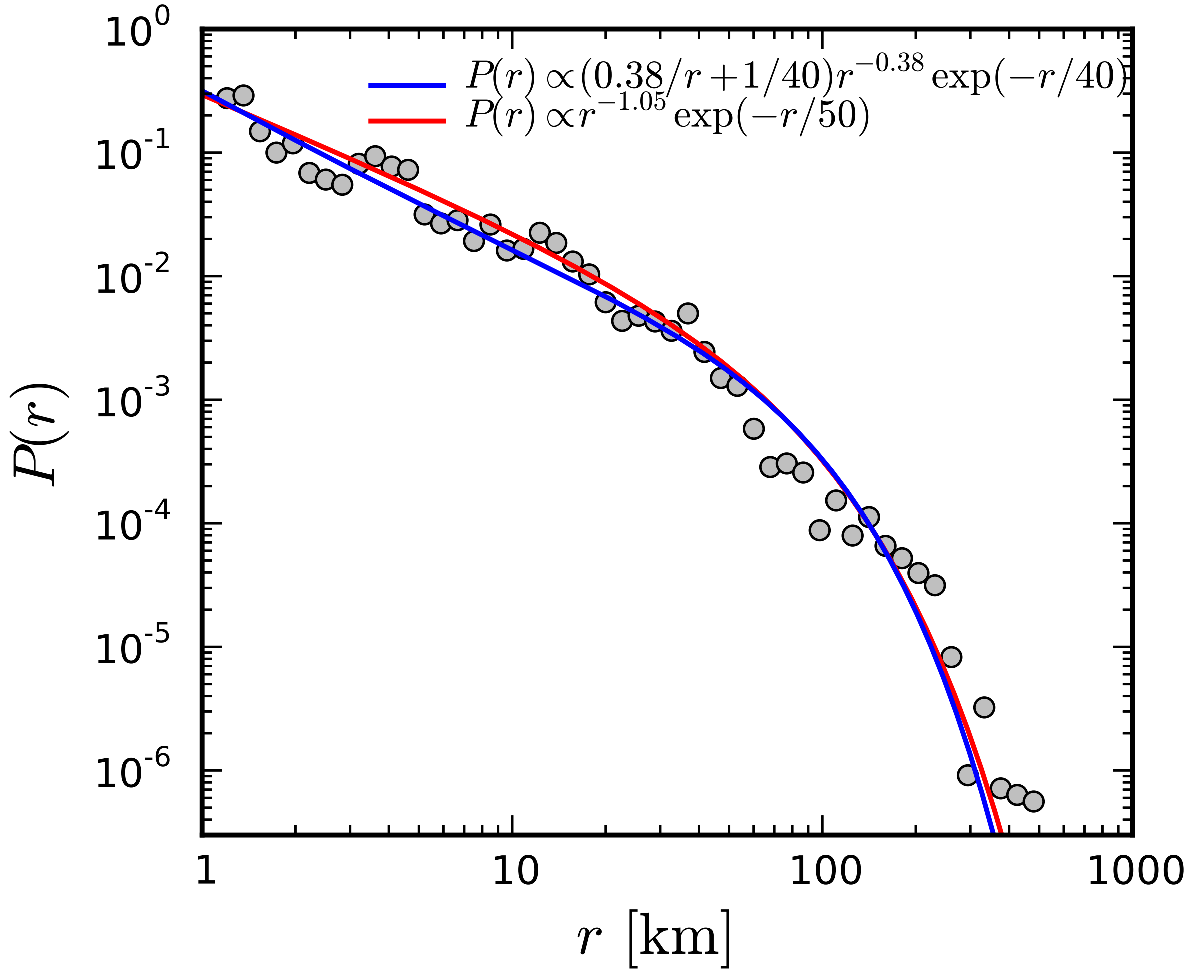

Figure S3: Comparison of the fitting potential of various probability density functions (red line and blue line) to the aggregated displacement distribution data. The data were binned using logarithmic binning method.

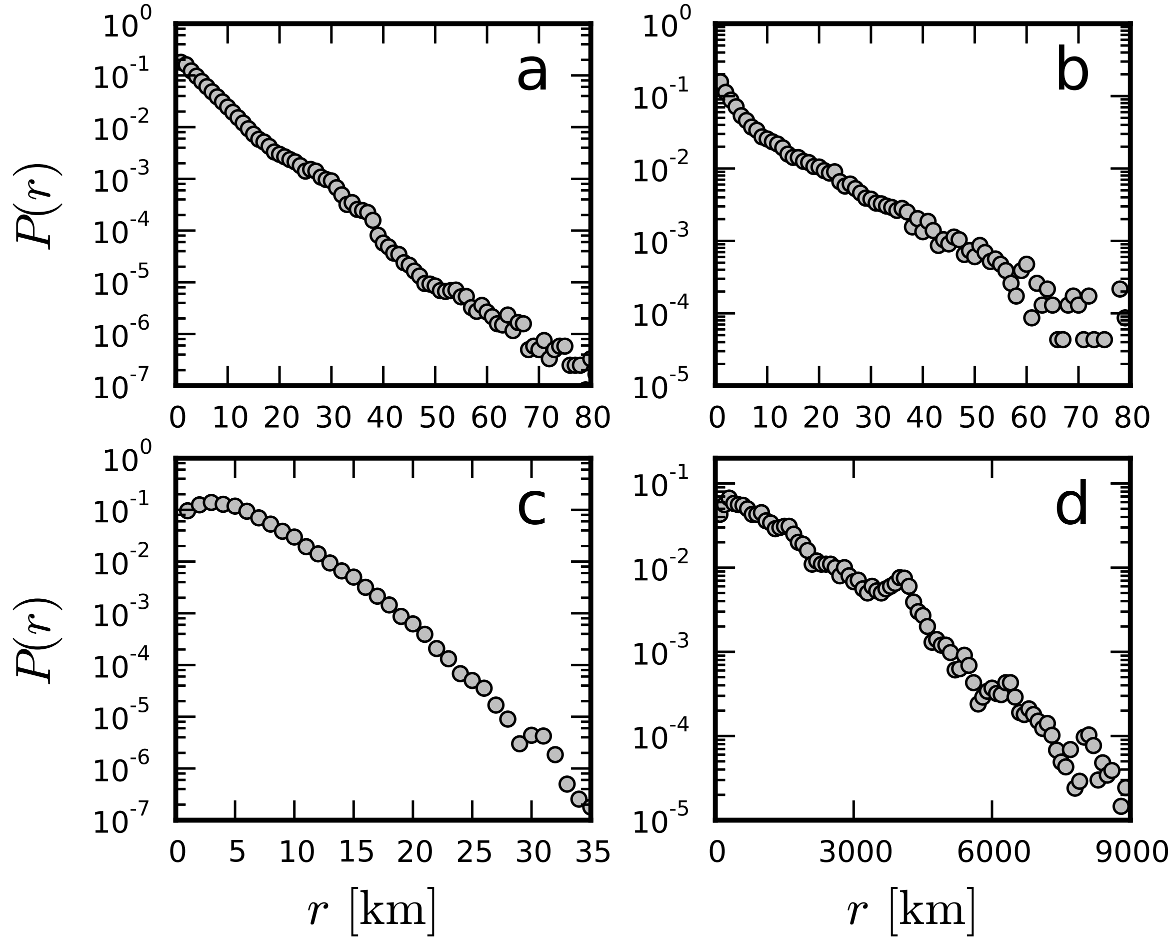

Figure S4: Displacement distributions for single mode of transportation (using probability density distribution). **a**. 12,028,929 taxi passenger trajectories in Beijing. **b**. 46,541 car trips in Detroit. **c**. 783,210 bus trips in Shijiazhuang. **d**. 205,534 air-flight passenger travels in US.

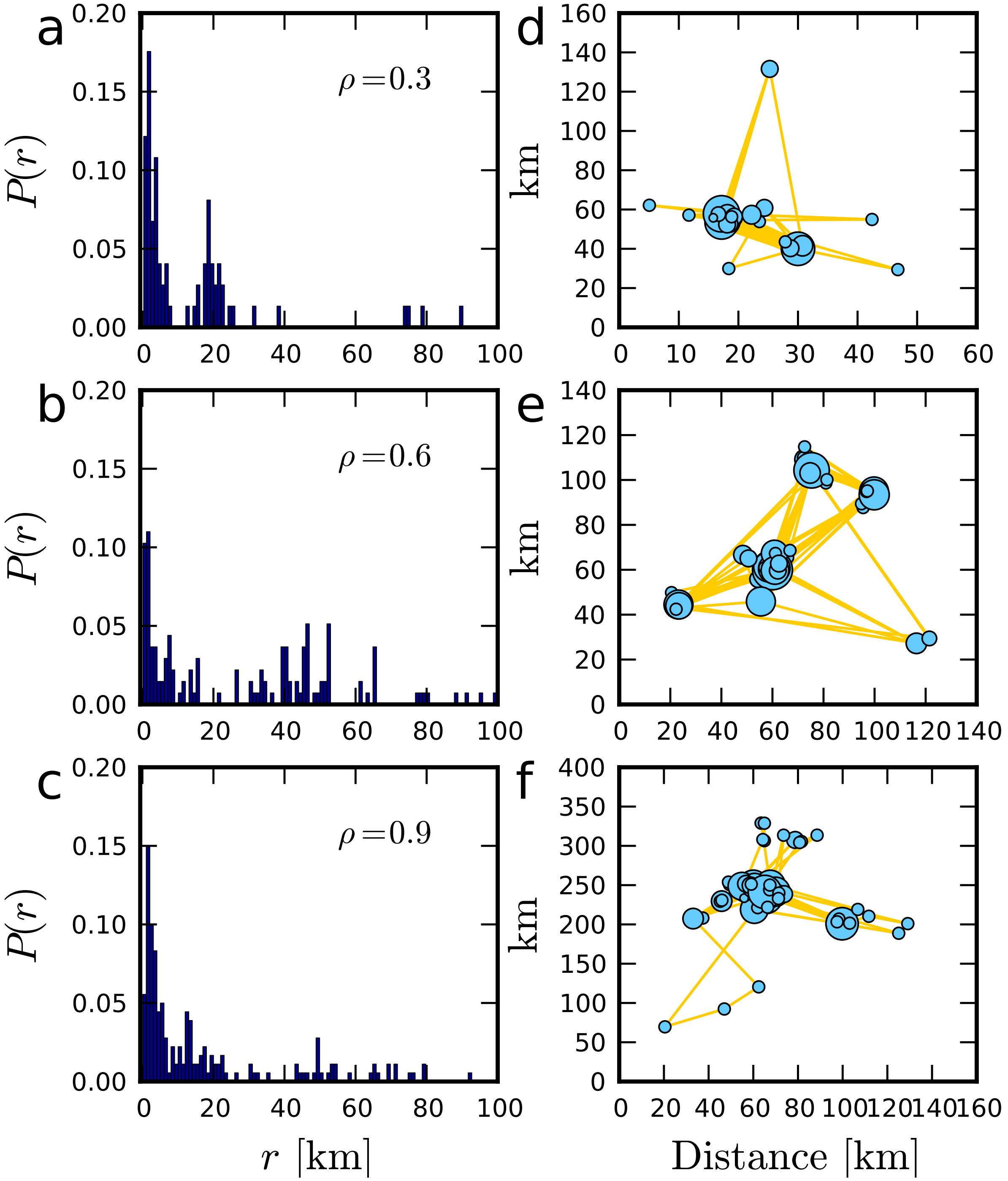

Figure S5: Individual mobility patterns generated by exploration and preferential return model proposed by Song, Koren, Wang and Barabási in *Nat. Phys.* **6**, 818 2010. **a-c**. Displacement distributions for three simulative individuals. ρ is a parameter to characterize the individual's preference of exploration new locations. **d-f**. Mobility networks for the three individuals, where the area of a node is proportional to its number of visits and the width of an edge is proportional to its weight.
